# A Comparison of Bioactive Metabolites, Antinutrients, and Bioactivities of African Pumpkin Leaves (*Momordica balsamina* L.) Cooked by Different Culinary Techniques

**DOI:** 10.3390/molecules27061901

**Published:** 2022-03-15

**Authors:** Petunia Mashiane, Tinotenda Shoko, Vimbainashe Manhivi, Retha Slabbert, Yasmina Sultanbawa, Dharini Sivakumar

**Affiliations:** 1 Department of Horticulture, Tshwane University of Technology, Pretoria 0001, South Africa; mashianep@tut.ac.za (P.M.); slabbertmm@tut.ac.za (R.S.); 2 Phytochemical Food Network Research Group, Department of Crop Sciences, Tshwane University of Technology, Pretoria 0001, South Africa; shokot@tut.ac.za (T.S.); manhivive@tut.ac.za (V.M.); 3 Agricultural Research Council Industrial Transformation Training Centre for Uniquely Australian Foods, Queensland Alliance for Agriculture and Food Innovation, The University of Queensland, Brisbane, QLD 4068, Australia; y.sultanbawa@uq.edu.au

**Keywords:** traditional leafy vegetables, phytochemicals, carotenoids, antioxidants, antinutrients

## Abstract

Prior to consumption, African pumpkin leaves (*Momordica balsamina* L.) are generally cooked. In this study, the effects of common household cooking methods (boiling, steaming, microwaving, stir-frying) on bioactive metabolites, carotenoids, antioxidant activity, antinutrients and inhibitory effects on α-glucosidase and α-amylase activities were examined. A set of 14 bioactive metabolites were identified in raw and cooked African leaves using UPLC-QTOF/MS. The results showed that the four different types of household cooking methods had different effects on the bioactive metabolomics profile of African pumpkin leaves. In comparison to raw leaves and leaves cooked in other methods, the concentrations of six phenolic compounds, rutin, cryptochlorogenic acid (4-caffeoylquinic acid), pseudolaroside A, isorhamnetin 3-O-robinoside, quercetin 3-galactoside, and trans-4-feruloylquinic acid, were highest in stir-fried leaves. Of all household cooking methods tested, stir-frying increased the content of lutein, β-carotene, and zeaxanthin by 60.00%, 146.15%, and 123.51%, respectively. Moreover, stir-frying African pumpkin leaves increased the antioxidant activity (DPPH and ABTS) and the inhibition of α-glucosidase and α-amylase. Compared to all four methods of household cooking, stir-frying reduced the antinutritive compounds compared to raw leaves. This work provides useful information to the consumers on the selection of suitable cooking methods for African pumpkin leaves.

## 1. Introduction

*Momordica balsamina* L., also known as African pumpkin or Balsam apple, is a wild traditional leafy vegetable of the Cucurbitaceae family [1]. African pumpkin leaves are mainly consumed in Africa and Asia; they have little commercial value. They usually grow as weeds alongside other main food crops and are cheap and easy to harvest [2]. Despite its nutritional and medicinal importance, there are still gaps in research regarding its nutritional value, especially for the leaves [3]. In comparison to conventional leafy vegetables, such as spinach, kale, and broccoli, African pumpkin leaves were found to contain higher levels of vitamins, minerals, and antioxidants [4]. Most rural populations in sub-Saharan Africa face food insecurity issues, malnutrition and micronutrient deficiencies; vitamin A deficiency is a common health concern. Therefore, traditional vegetables such as African pumpkins are the cheapest and contain readily available sources of several important micronutrients for food security [5]. The leaves of the African pumpkin are promoted as a protein supplement for diets based on cereal in poor rural communities [6].

The United States Department of Agriculture [USDA] guidelines [7] suggest individuals should consume 1.5 cups [∼237 g] of raw or cooked vegetables or two cups of raw leafy greens. Traditionally, African pumpkin leaves are cooked as a relish to accompany maize meal porridge (the staple starch), or the leaves and green fruits are cooked with crushed groundnuts as gravy [8]. African pumpkin leaves are commonly consumed in their cooked form (thermal processing methods), including boiling, steaming, microwaving, and stir-frying [9]. Cooking denatures browning enzymes, reduces or eliminates bitterness from vegetables, and removes acids [10]. Cooking changes the chemical composition of vegetables profoundly, changing their bioavailability and amount of bioactive compounds [4,11]. According to Managa et al. [12], cooking can enhance the availability of phenolic compounds and the antioxidant capacity of vegetables. Additionally, dietary phenolic compounds inhibited carbohydrate enzymes, including α-amylase and β-glucosidase, and acted as appropriate anti-glycaemic agents [13]. The aforementioned enzymes play a vital role in controlling blood glucose levels and obesity by reducing the absorption of glucose in the intestine [9]. Our previous studies have shown that steaming leaves of African pumpkin decreased the loss of chlorophyll content while increasing the level of carotenoids by 22.65% compared to boiling, microwaving and stir-frying [14]. Additionally, steaming reduced the loss of total phenolic content and retained the antioxidant activity in both African pumpkin and pumpkin leaves compared to other methods.

It has been reported that African pumpkin leaves contain bioactive metabolites that may assist in reducing oxidative stress in the human body [1]. Postprandial hyperglycemia results from pancreatic α-amylase hydrolyzing carbohydrates to dextrin, which is hydrolysed further to glucose by intestinal α-glucosidase. Inhibiting these two enzymes is an important strategy for managing type 2 diabetes [15]. Our previous study examined the effects of household cooking methods (stir-frying or boiling) on changes in bioactive metabolites, antioxidant capacity, release and accessibility of β-carotene, and inhibition of inhibitory activity against α-amylase and α-glucosidase enzymes during in vitro digestion of African pumpkin leaves [16]. Based on the study, it was clear that dialysable fractions of stir-fried leaves possessed the greatest inhibitory activity against both α-amylase and α-glucosidase enzymes, as well as acarbose [16]. The bioaccessibility of phenolic compounds and beta carotene from African pumpkin leaves during digestion depends on the type of household cooking method used [16]. While the previous study recommended stir-frying African Pumpkin leaves over boiling or raw leaves for the release and bioavailability of most bioactive phenolic metabolites, it did not compare that method to other commonly used food preparation methods such as steaming and microwaving [16]. In addition, there are no previous reports on the impact of different cooking methods, including steaming, microwaving with stir-frying and boiling of African pumpkin leaves on changes in phenolic compounds, different carotenoid components, antioxidant properties and antinutritive compounds. Phytates, oxalates and alkaloids are common antinutritive compounds found in green leafy vegetables [17].

Tannins form complexes with zinc and proteins and, in turn, make them insoluble, preventing them from being digested and absorbed [18]. Oxalates bind with dietary calcium and prevent it from being absorbed [12,18]. Phytates reportedly inhibit mineral absorption, lower the bioavailability of metal ions, such as zinc ions, and affect protein and starch digestion [18]. Different cooking techniques reportedly reduce the antinutritive content of traditional leafy vegetables. Consequently, it is imperative to consider this when selecting the most suitable cooking process to preserve the antioxidants and phytochemicals in cooked leafy vegetables.

This study is important for the consumers and the chefs involved in African cuisine to standardise a suitable household cooking method for food preparation. 

Therefore, the purpose of this study was to examine the effect of different household cooking processes on phenolic compounds, carotenoid components, antioxidant properties, activity against carbohydrate hydrolysing enzymes (α-glucosidase and α-amylase) and antinutritive compounds in African pumpkin leaves.

## 2. Results and Discussion

### 2.1. Changes in Bioactive Metabolites during Different Household Cooking

A set of 14 major bioactive metabolites detected in raw and cooked African leaves by UPLC-QTOF/MS as described previously by Mashiane et al. [16]. The Supplementary Figures S1–S14 includes the UV spectrum and the MS and MS/MS fragmentation pattern of the identified bioactive metabolites. 

Metabolites included flavonoid glycosides (35.7%), quinic acid derivatives (28.6%), cinnamic acids and derivatives (14.3%), hydroxycinnamic acid glycosides (7.1%), O glycosides (7.1%) and phenolic glycoside (7.1%). The MS spectrum of peak 1 had a parent ion at *m/z* 205[M-H]^−^ and the second-order mass spectrum of peak 1 had a characteristic dehydrated quinic acid fragment at *m/z* 173. In the MS/MS spectrum of peak 2, a base peak ion was found at *m/z* 137[M-H-162]^−^, which was due to the cleavage of a hexoside. A fragment ion at *m/z* 93[M-H-162-44]^−^ was found as the result of subsequent cleavage of the hexoside from the parent ions and subsequent decarboxylation of the aglycone moiety. Mashitoa et al. [19] observed this fragmentation pattern for pseudolaroside A. The tentative identification of peak 2 was pseudolaroside A. The second-order spectrum of peak 3 had a characteristic fragment ion at *m/z* 163[hydroxycinnamic acid-H]^−^ [20]. Peak 3 was confirmed tentatively as melilotoside. Peak 4 has a parent ion in its MS spectrum at *m/z* 353[M-H]^−^; in its second-order spectrum, peak 4 exhibited characteristic quinic acid fragments at *m/z* 191 (deprotonated quinic acid) and *m/z* 173 (dehydrated quinic acid). Fang et al. [21] identified 4-caffeoylquinic acid based on its fragmentation pattern and the base peak ion at *m/z* 173. Peak 4 was tentatively identified as 4-caffeoylquinic acid (cryptochlorogenic acid). The first-order spectra of peaks 5 and 6 showed a parent ion at *m/z* 337 [M-H]^−^ due to deprotonation. In the MSE spectrum, the 2 compounds exhibited a characteristic quinic acid fragment ion at *m/z* 191 and a coumaric acid fragment at *m/z* 163[M-H-174]^−^ due to loss of a quinic acid fragment ion [21]. Clifford et al. [22] observed compounds with similar peaks and classified them as p-coumaroylquinic acids. Moreover, there was a fragment ion present in both peaks at *m/z* 173[M-H-163]^−^ due to the loss of coumaric acid. Based on the studies of Clifford et al. [22], both peaks were identified as isomers of 4-coumaroylquinic acid. Peak 5 was thus identified as cis-4-coumaroylquinic acid, and peak 6 was identified as trans-4-coumaroylquinic acid based on elution times [23]. In the MS spectrum, peaks 7 and 9 had molecular ions at *m/z* 367. In their MS/MS spectra, a fragment ion characteristic of dehydrated quinic acid was observed at *m/z* 173[M-H-177-18]^−^ due to subsequent cleavage of the feruloyl fragment and dehydration of the quinic acid fragment. Peaks 7 and 9 are identified as 4-feruloylquinic acid isomers. Based on elution times, peak 7 was identified as cis-4-feruloylquinic acid and peak 9 as trans-4-feruloylquinic acid [23]. The first-order mass spectra of peaks 8 and 10 showed molecular ions at *m/z* 609 and *m/z* 463, respectively. In their second-order mass spectra, both peaks exhibited a base peak ion at *m/z* 300, which has been shown to be indicative of quercetin derivatives [24]. Other secondary fragments were obtained in both peaks due to fragmentation of quercetin at *m/z* 271[M-H-308-28]^−^ from subsequent cleavage of a diglycosyl unit and a carbon monoxide parent ion at *m/z* 179, which was due to retrocyclisation following fission on the quercetin C ring. A carbon monoxide unit was cleaved from the *m/z* 179 fragments to produce the fragment ion at *m/z* 151. Based on similar fragmentation patterns, peaks 8 and 10 were tentatively identified as rutin [19] and quercetin galactoside, respectively [23]. The MS spectrum of peak 11 exhibited a deprotonated molecular ion at *m/z* 593[M-H]^−^. In the MSE spectrum, a base peak ion consistent with kaempferol was observed at *m/z* 285[M-H-309]^−^ due to cleavage of a diglycosyl moiety [25]. A secondary peak consistent with the rhamnose moiety was observed at *m/z* 163[M-H-285-147]^−^ due to subsequent loss of a kaempferol fragment and a glycosyl residue. The tentative identification of peak 11 was as kaempferol-O-rutinoside (nicotiflorine). In the MS spectrum, peak 12 exhibited a parent ion at *m/z* 623[M-H]^−^. In the MS/MS spectrum, a base peak ion consistent with the isorhamnetin aglycone skeleton was observed at *m/z* 315[M-H-162-147]^−^ due to subsequent loss of a rhamnosyl and a galactosyl residue [26]. Furthermore, there was a quercetin fragment observed at *m/z* 300 due to the demethylation of the isorhamnetin aglycone. As such, peak 12 was tentatively identified as isorhamnetin 3-O-robinoside (keioside). Peak 13’s MS/MS fragmentation pattern exhibited a base peak ion consistent with a methoxylated rhamnetin aglycone at *m/z* 314[M-H-162]^−^ from cleavage of the glycosyl residue [27]. Cleavage of a methyl group and a methoxy group from the methoxylated rhamnetin gave the quercetin fragments observed at *m/z* 300 and *m/z* 285, respectively. As such, the tentative identification of peak 13 was as rhamnetin-3-*O*-glucoside. The MS/MS fragmentation pattern of peak 14 exhibited a fragment characteristic of a diglycoside at *m/z* 325[M-H-105]^−^ due to loss of a phenethyl residue. Cleavage of the diglycoside residue gave a glycoside residue at *m/z* 161; thus, the tentative identification of peak 14 was as phenethyl rutinoside. The Supplementary Figures S1–S14 includes the UV spectrum and the MS and MS/MS fragmentation pattern of the identified bioactive metabolites were performed as shown by Masiane [16].

The changes in bioactive metabolites after processing African pumpkin leaves using different cooking methods are provided in Table 1. The most abundant bioactive metabolite identified with UPLC-QTOF/MS was *cis*-4-feruloylquinic acid (1505.20 mg kg^−1^). In comparison to raw leaves, all four methods of household cooking reduced the levels of *cis*-4-feruloylquinic acid and methylquinic acid. Despite steaming, boiling, and microwave cooking destroying *cis*-4-coumaroyl quinic acid and *trans*-4-coumaroyl quinic acid, stir-frying increased their concentrations in African pumpkin leaves in comparison to raw leaves. This can be attributed to stir-frying causing matrix softening due to cell wall degeneration rendering phytochemicals more accessible as compared to raw leaves [28]. In comparison to raw leaves, stir-frying and steaming, respectively, resulted in the loss of 91.67% and 63.64% of methylquinonic acid. Meanwhile, microwave cooking resulted in a higher loss of *cis*-4-coumaroyl quinic acid (95.55%), *trans*-4-coumaroylquinic acid (91.03%), *cis*-4-feruloylquinic acid (98.55%) and trans-4-feruloylquinic acid (99.44%) in comparison to the raw leaves. In African pumpkin leaf samples, steaming, microwaving, and boiling reduced the losses of cis-4-coumaroyl quinic acid, methylquinic acid, and *trans*-4-coumaroyl quinic acid, respectively, in comparison to raw leaves. According to Sergio et al. [28], losses in the levels of individual phenolic constituents are a result of thermal degradation and water leaching, leading to a reduction in their concentrations. However, 4-caffeoylquinic acid increased with steaming, boiling, and stir-frying but decreased with microwave cooking compared to raw leaves. Conversely, 4-caffeoylquinic acid increased with steaming, boiling and stir-frying and decreased with microwave cooking in comparison to raw leaves. In steaming, boiling, and stir-frying, 4-caffeoylquinic acid may increase due to the breakdown of the cellular wall during cooking and the enhanced availability for extraction of phenolic metabolites. Furthermore, the increase in 4-caffeoylquinic acid after cooking may be due to hydrolysis reactions and other reactions such as the Maillard reaction products leading to the substantial redistribution of phenolic metabolites. A similar significant increase in caffeoylquinic acids was reported by Ferracane et al. [29] in fried artichoke leaves in comparison to raw and other cooking methods. Managa et al. [30] reported similar findings of 4-caffeoylquinic acid during all cooking methods, with the highest levels detected during stir-frying. 

To compare the means of the cooking treatments, the least significant difference test (LSD) was used, with *p* < 0.05.

High temperatures also increased the level of 4-*O*-caffeoylquinic acid due to the isomerisation and transformation of 5-caffeoylquinic acid [31] in artichokes [29]. Wang and Ho [31] reported that 5-caffeoylquinic acid could be hydrolysed during thermal treatment to produce caffeic acid. However, our results contradict theirs, suggesting that boiling, microwaving, or steaming may have caused caffeic acid to leach out. There was a discrepancy regarding the retention of quinic acid derivatives reported in the literature during the microwave cooking of chicory [32] and nightshade [30] leaves, respectively, while our study observed a decrease in their content. The pH of the food matrix, as well as the temperature and time, affect the transesterification of caffeoylquinic acid derivatives [30]. Quercetin 3-galactoside, phenethyl rutinoside, rhamnetin 3-*O*-glucoside, pseudolaroside A, kaempferol-*O*-rutinoside and isorhamnetin-3-*O*-robinoside decreased significantly during steaming, microwaving, or boiling in comparison to the raw leaves, while rutin lost more than 90% during boiling and microwaving compared to the raw leaves. Conversely, β-d-glucosyl-2-coumarate was reduced to 91.14% and 59.66% during microwaving and stir-frying, respectively.

Stir-frying increased quercetin-3-galactoside, rhamnetin 3-*O*-glucoside, rutin, pseudolaroside A, kaempferol-*O*-rutinoside and isorhamnetin-3-*O*-robinoside significantly more than all other 4 methods employed in this study. Moreover, steaming increased rutin and β-d-glucosyl-2-coumarate. Cheng et al. [33] also reported that boiling and microwaving decreased the content of phenolic metabolites of three varieties of pak choi (*Brassica rapa* subsp. Chinensis) due to leaching and degradation of water-soluble bioactive compounds. A flavonoid’s thermostability depends on its glycosylation and acylation status, and the thermal processing mode will determine the thermostability [34]. Boiling and microwave treatment deacylates and deglycosylates flavonoid glycosides, resulting in non-acylated flavonoid glycosides. Most likely, there were no compounds detected because they leached out [35,36]. According to the above-mentioned results, most of the individual phenolic compounds detected by UPLC-QTOF/MS in African pumpkin leaves showed a similar increase in content after stir-frying and a decrease after boiling or microwaving. Stir-fried African pumpkin leaves had more bioavailability of many phenolic metabolites than raw African pumpkin leaves. Due to the destruction of cell walls and other subcellular components associated with over stir-frying, there could have been a release of most of the phenolic compounds [37]. Further, the increase in phenolics after stir-frying African pumpkin leaves could be due to the enhanced availability for extraction and a more efficient release of flavonoid compounds from intracellular proteins and altered cell wall structures [38]. Similar results have been reported by Hossain et al. [39], who reported an increase in the flavonoid content of stir-fried green amaranthus leaves and Indian spinach. Olive oil, a nonpolar medium used during stir-frying, could have prevented the loss of phenolic compounds due to lack of diffusion or migration into the medium [36,37].

### 2.2. Multivariate Analysis

**Figure 1:**
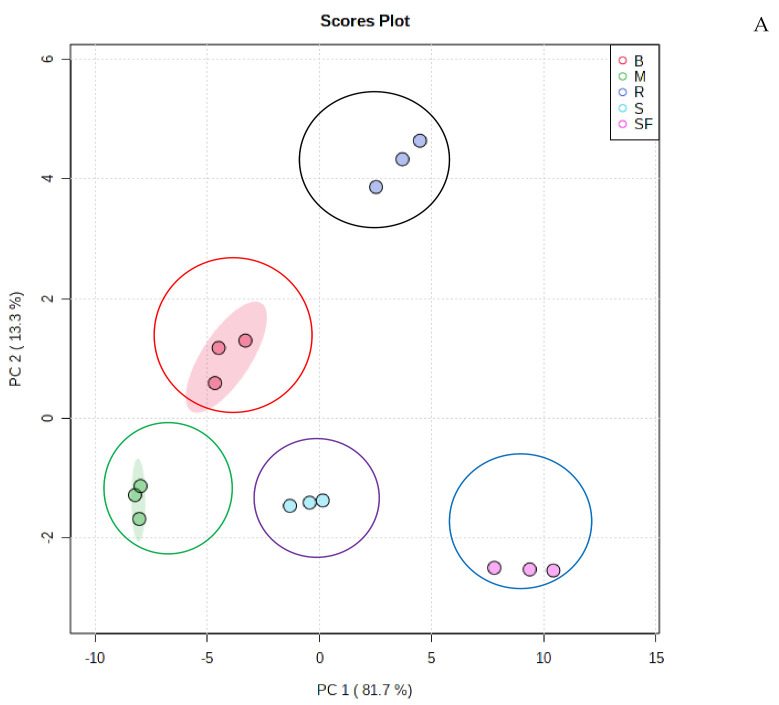

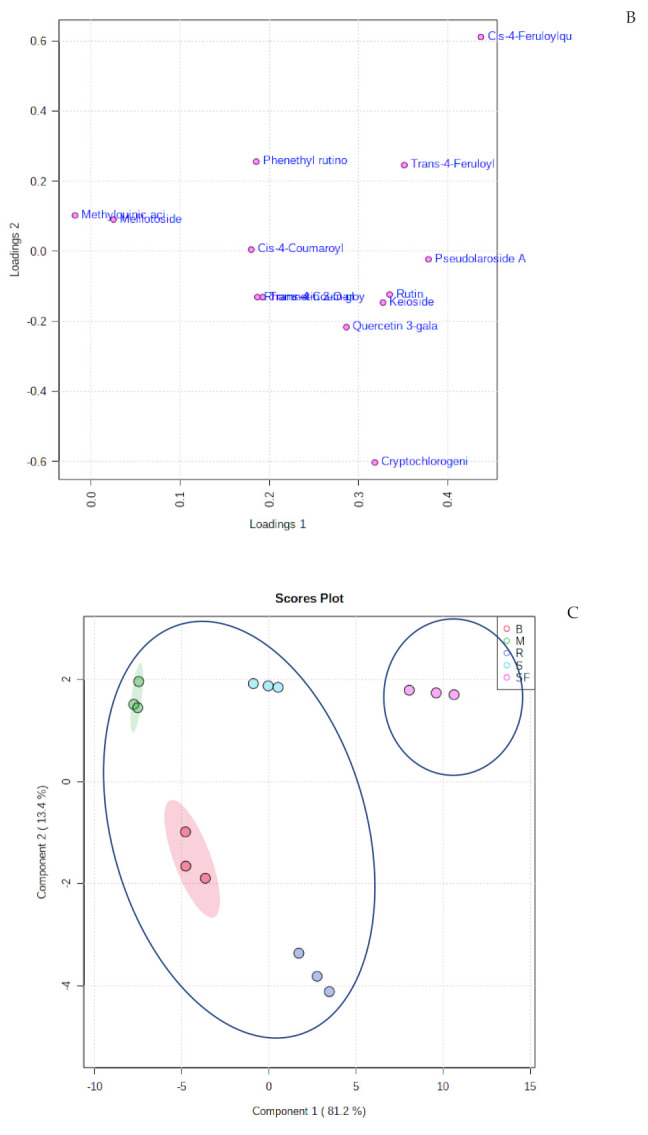

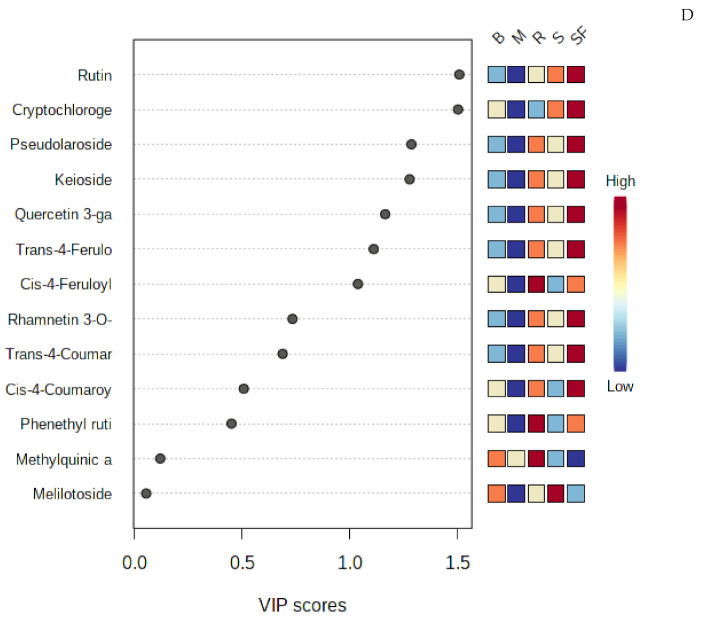

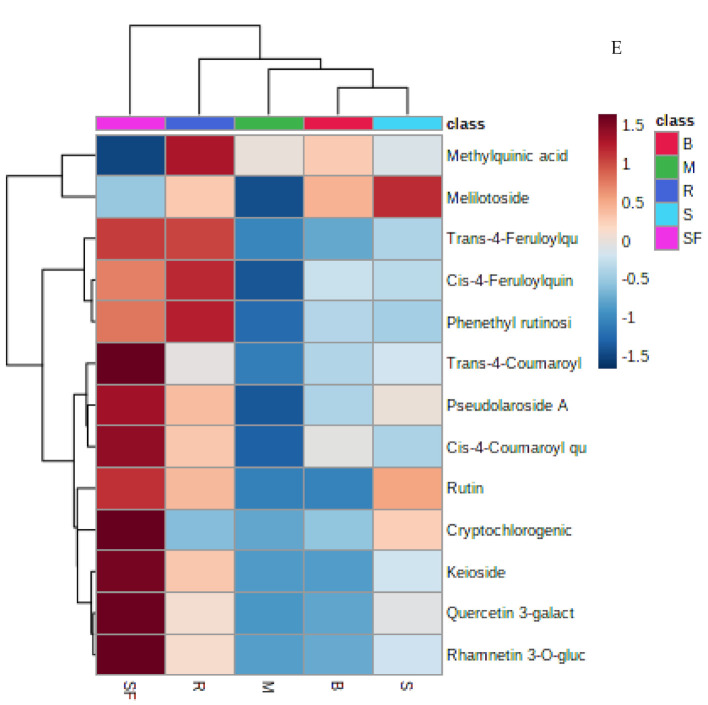
Statistical analyses of bioactive metabolites by Metaboanalyst 4.0 software. (**A**) Unsupervised PCA score plot of phenolic compounds of *Momordica balsamina* leaves generated by UPLC-QTOF/MS analysis showing the separation of five clusters. R: raw leaves; M: microwaved leaves; S: streamed leaves; B: boiled leaves; SF: stir-fried leaves. (**B**) The PC 1 and PC2 loadings of bioactive metabolites. (**C**) Supervised PLS-DA score plot of bioactive metabolites. (**D**) VIP scores of bioactive metabolites in PLS-DA. (**E**) Heat map. The colored areas on the map correspond to concentrations of different bioactive metabolites. Bioactive metabolites are arranged in rows, and different household cooking techniques are in columns. The red color indicates high levels, and the blue color indicates low levels.

A principal component analysis (PCA) method, using the data generated by UPLC-QTOF/MS, determined the differences in bioactive metabolites between different techniques of household cooking and discriminated between these techniques (Figure 1A,B). According to a PCA plot, the leaves of the African pumpkin were grouped into five distinct districts depending on which cooking method was used (Figure 1A). We used the data on phenolic metabolites to discriminate between the household cooking methods based on their greater impacts on phenolic metabolites. PC1 explained 81.7% of the variance, with African pumpkin leaves from stir-fried and raw leaves positioned along with positive PC1 scores and boiled, steamed, and microwaved leaves along with negative PC1 scores (Figure 1A). PC2 accounted for 13.3% of the variance, with raw African pumpkin leaves positioned along the positive PC2 and stir-fried leaves along the negative PC2. The score plot in Figure 1A shows that both PCs (PC1 & PC2) together accounted for more than 95% of the variance in the data. Consequently, Figure 1A indicates reliable discrimination among different household cooking methods of African pumpkin leaves and raw leaves. In the PCA plot, the leaves of the African pumpkin were classified into four distinct districts depending on the cooking method. The compounds 4-caffeoylquinic acid, isorhamnetin rutin, quercetin 3-galactoside, pseudolaroside A, trans-4-coumaroylquinic acid and rhamnetin-3-*O*-glucoside were the main phenolic metabolites positioned most distantly with positively influencing PC1 (Figure 1B), suggesting that these compounds are responsible for the discrimination of stir-fried leaves from the other three household cooking methods and the raw leaves. Additionally, β-d-glucosyl-2-coumarate and methylquinic acid were positioned at PC1 (negative) (Figure 1B) and influenced the separation of the boiled and microwaved samples from the rest. Among the compounds characterised in Figure S1, *cis*-4-feruloylquinic acid, placed at the furthest distance, positively affected PC2 (Figure 1B) for separating raw leaves from stir-fried leaves.

In PLS-DA, multiple linear regression techniques determine which direction maximum covariance between a dataset (X) and class membership (Y) will appear. Using PLS-DA, the basis for the classification of the different types of household cooking and raw African pumpkin leaves was on their phenolic compounds. On the score plot of phenolic compounds, raw, steamed, boiled, microwaved and stir-fried African pumpkin leaves were clearly categorised according to PC1 and PC2 components. PLS-DA plots showed two distinct groups of stir-fried leaves, while the rest of the raw, steamed, boiled, microwaved leaves were grouped in another cluster. The cumulative contribution rate was 94.6% (Figure 1C).

By adding the squares of the PLS loadings across all dimensions and adding the weighted sum of the PLS regression coefficients, VIP scores are calculated [40]. The VIP score is used to rank the metabolites. In order to interpret the results in the most meaningful way, only the best metabolites are considered [40]. Using the VIP score analysis, we evaluated the influence of phenolic compounds on the differentiation of African pumpkin leaf cooking methods. Figure 1D illustrates that all seven phenolic compounds (rutin, 4-caffeoylquinic acid (cryptochlorogenic acid), pseudolaroside A, isorhamnetin-3-*O*-robinoside (keioside), quercetin-3-galactoside, and trans-4-feruloylquinic acid) accounted for a significant contribution (VIP > 1). In this study, the use of these phenolic compounds was to differentiate raw, steamed, boiled, and stir-fried African pumpkin leaves. Rutin and 4-caffeoylquinic acid (cryptochlorogenic acid) showed very high VIP scores of 1.5, and these 2 compounds distinguished the stir-fried leaves from the other household cooking methods. In raw and cooked African pumpkin leaves, heat map visualization, based on color intensity, can directly correlate to the quantitative estimation of phenolic compounds (Figure 1E). Furthermore, the heat map revealed that phenolic compounds were more abundant in stir-fried African pumpkin leaves than in raw leaves, boiled leaves, microwaved leaves, or steamed leaves. From the PLS-DA loading, VIP plot, and heat map, a few main phenolic constituents of stir-fried African pumpkin leaves and other cooking methods, including the raw leaves, were highlighted as potential discriminant markers. Rutin (VIP score 1.511) and 4-caffeoylquinic acid (cryptochlorogenic acid) (VIP score 1.512) were noted as the marker candidates that contributed greatly to the separation of stir-fried leaves from the other cooking methods and raw leaves.

The most abundant compounds in different household cooking methods (B, S, SF, M and R) are represented by their *m/z* values and retention times (min). Variables ranging from low to high numbers are scored according to their importance. On the right, the colored boxes indicate the relative concentration of the corresponding metabolites. A red color indicates a high level, and a blue color indicates a low level. Colors are used to represent two-dimensional tables of numbers in heat maps. Heat map plotting is a popular technique used to visualize changes in multivariate data [41]. A heat map structure was developed from the different concentrations of phenolic compounds across all samples in conjunction with this analysis. The hierarchical cluster analysis confirmed that there were two major clusters in the PLS-DA plot (Figure 1C), as indicated by the cladogram at the top of Figure 1E. On the heat map, each row of data relates to the phenolic compounds, and the type of household cooking method represents the column of a colour block, with darker red boxes for higher levels and darker blue boxes for lower levels of phenolic compounds [16].

### 2.3. Carotenoid Profile in African Pumpkin Leaves (Momordica balsamina *L.*)

Table 2 shows the carotenoid profiles of African pumpkin leaves subjected to different cooking techniques in comparison to raw leaves. In comparison to raw leaves, African pumpkin leaves subjected to different household cooking techniques had the highest carotenoid content. African pumpkin leaves are rich in lutein, one of three carotenoid components. Zeaxanthin, found to be a minor component of cooked and raw African pumpkin leaves, only ranged from 0.64 to 3.20 mg100 g^−1^. In comparison to raw leaves, stir-frying increased the content of lutein, β-carotene, and zeaxanthin by 60%, 146.15%, and 123.51%, respectively. Stir-frying increased the retention of β-carotene in all vegetables two to three-fold in comparison to raw Chinese cabbage (*Brassica pekinensis* var. cephalata) and swamp cabbage (*Ipomoea aquatica*) [5]. Carotenoids are oil-soluble; as such, the use of oil in stir-frying increased the extraction of carotenoids from the leaves, resulting in the higher carotenoid levels observed in stir-fried leaves [5]. Due to changes in tissue morphology that occur during stir-frying, extracting solvents might have entered cells more readily, enhancing the provision of β-carotenes [42]. This increase in lutein is due to its liberation from protein or lipid binding forms to become free lutein [42]. In addition, Zib and Nisar [42] suggested that lutein from oil mediums might enter the leaf matrix and contribute to its increased levels. Furthermore, thermal treatments can result in trans/cis- isomerization, such as the formation of 13-cis carotene or the formation of different carotenoids by-products [43]. Additionally, microwaving reduces lutein content by 22.21% and β-carotene by 11.93% in comparison to raw leaves. However, Akdas and Bakkalbaşi [5] report that stir-frying reduced the number of carotenoids in kale by 28.2%. Next to stir-frying, steaming preserved the carotenoid components and total carotenoid content better than boiling and microwaving. Similar to our results, Pellegrini et al. [44] reported that boiling and microwaving reduced total carotenoid content in broccoli. Due to disruption of the cell wall and chloroplasts during boiling or microwave cooking, carotenoids leached into the water. The volume of water used for cooking was an influencing parameter affecting carotenoid retention [43]. The current recommendation for retinol activity equivalents (RAE) is 900 mg for men and 700 mg for women per day. Stir-fried African pumpkin leaves can provide about 0.09 g of RAE (retinol activity equivalents) for adults, 0.12 g for women and 0.21 g for children.

### 2.4. Antioxidant Capacity and Antidiabetic Activity

There are two types of free radicals in this compound: DPPH, which has an unpaired electron on one atom of the nitrogen bridge, and ABTS, which is another free radical. These two methods estimate the free radical scavenging activities of antioxidants [45]. The IC_50_ value is the concentration needed for 50% inhibition and determines the strength of the inhibitor [19,46]. The antioxidant activities of African pumpkin leaves cooked by adopting four different house hols cooking methods are given in Table 3. According to DPPH and ABTS^+^ assays, stir-fried African pumpkin leaves demonstrated the highest antioxidant activity in comparison to raw leaves and those cooked by boiling, steaming, or microwaving. The antioxidant activities of different household cooking methods were in the following order, from highest to lowest: stir-frying > raw > steaming > boiling > microwaving. Furthermore, Hussain et al. [39] reported that frying increased amaranthus’ radical scavenging ability by 111.98% compared to raw leaves. During stir-frying, cells and subcellular compartments are destroyed by heat, and antioxidant components are released and mixed with oil. They then either remain on the surface of the leaves, contribute to the production of stronger antioxidants by thermal chemical reactions, facilitate the inactivation of oxidative enzymes by thermal treatment, or encourage the formation of new antioxidants and other compounds, such as Maillard reaction products that are antioxidants [47]. It is possible that all these events contributed to the observed antioxidant activity in the stir-fried African leafy vegetables. Additionally, boiling the small *Momordica charantia* (bitter gourd) for 20 min reduced its scavenging activity [47]. According to Zhao et al. [48], cooking changes in antioxidant activity are the result of changes in phytochemicals. Based on a regression correlation test, we analyzed the relationship between individual phenolic compounds or carotenoids in African pumpkin leaves and their antioxidant activity. Rutin (R^2^ = 91) showed the highest correlation with DPPH scavenging activity, followed by kaempferol-O-rutinoside (R^2^ = 90), pseudolaroside A (R^2^ = 85), trans-4-feruloylquinic acid (R^2^ = 82), cis-4-coumaroylquinic acid (R^2^ = 74), isorhamnetin-3-O-robinoside (R^2^ = 70), trans-4-coumaroylquinic acid (R^2^ = 66), quercetin 3-galactoside (R^2^ = 66), rhamnetin-3-O-glucoside (R^2^ = 65), cis-4-feruloylquinic acid (R^2^ = 61), phenethyl rutinoside (R^2^ = 60), and 4-caffeoylquinic acid (R^2^ = 60). Lutein was the only carotenoid to show a positive relationship with ABTS activity. The highest correlation for phenolic compounds was found for rutin (R^2^ = 75), followed by trans-4-feruloylquinic acid (R^2^ = 63), kaempferol-O-rutinoside (R^2^ = 61), cis-4-feruloylquinic acid (R^2^ = 60), and phenethyl rutinoside (R^2^ = 57).

Inhibition of α-glucosidase and α-amylase activity by the leaf extracts of African pumpkin leaves cooked by different methods is given in Table 3. Stir-fried African pumpkin leaves showed higher α-glucosidase and α-amylase activities than raw leaves or leaves cooked by boiling, steaming and microwaving. Raw and cooked African pumpkin leaves also showed high levels of α-glucosidase activity in comparison to the commercial inhibitor, acarbose. Similarly, nightshade, Chinese cabbage and cowpea leaf extracts inhibited α-glucosidase activity more than raw leaves or commercial inhibitors, such as acarbose [12,13].

Moreover, microwaving followed by boiling reduced the activity of α-glucosidase and α-amylase in comparison to steaming and stir-frying. Boiling also reduced the ability of *Momordica charantia* (small bitter gourd) leaf extracts to inhibit α-amylase activity [15]. In contrast, Subramaniam et al. [15] showed that microwaved small bitter gourd had the best ability to inhibit the activity of the α-glucosidase enzyme at 10 min (17.5%), while boiled samples showed the most inhibition at 5 min (14.8%). Microwave treatment of *Moringa* oleifera (drumstick) leaves for 20 min inhibited enzyme activity by the highest percentage (21.6%). Phenolic compounds, including rutin (R^2^ = 0.76), kaempferol-*O*-rutinoside (R^2^ = 0.75), *trans*-4-feruloylquinic acid (R^2^ = 0.72), cis-4-coumaroyl quinic acid (R^2^ = 0.66), *cis*-4-feruloylquinic acid (R^2^ = 0. 64), phenethyl rutinoside (R^2^ = 0.60, isorhamnetin-3-*O*-robinoside (R^2^ = 0.54), *trans*-4-coumaroylquinic acid (R^2^ = 0.53), and quercetin-3-galactoside (R^2^ = 0.50) correlated with α-glucosidase activity. Moreover, *trans*-4-feruloylquinic acid (R^2^ = 0.83), kaempferol-O-rutinoside (R^2^ = 0.81), rutin (R^2^ = 0.80), phenethyl rutinoside (R^2^ = 0.70), *cis*-4-feruloylquinic acid (R^2^ = 73), *cis*-4-coumaroylquinic acid (R^2^ = 62), isorhamnetin-3-*O*-robinoside (R^2^ = 0.54), and *trans*-4-coumaroyl quinic acid (R^2^ = 51) correlated with α-amylase activity. The OH groups at positions 3 (ring C), 7 (ring A), 4 and 5 (ring B) play a crucial role in the inhibition of α-glucosidase and α-amylase activities of polyphenols. Furthermore, the total number of hydroxyl groups, C-2-C-3 double bonds and C-4 ketonic functional groups all contribute to the antidiabetic effect [49]. Thus, the results indicated that phenolic metabolites could manage postprandial glycaemia. 

### 2.5. Antinutritive Compounds

Table 4 presents the changes in antinutrient compounds in African pumpkin leaves during different cooking methods. African pumpkin leaves were significantly (*p* < 0.05) higher in tannins (165.20 mg 100 g^−1^), phytates (41.37 mg 100 g^−1^) and alkaloids (35.00 mg 100 g^−1^) than those leaves subjected to different household cooking techniques (Table 4). Boiling and microwaving reduced tannin content by more than 60% and phytate content by more than 50% in comparison to raw African pumpkin leaves. Furthermore, the alkaloids in raw leaves were reduced by 88.46% through boiling and 57.49% by microwaving. Following the destruction of the cell walls of the leaves by microwave and boiling, it is likely the antinutritive compounds leached out into the boiling water. After boiling pumpkin leaves, Mashitoa et al. [46] reported reduced tannin concentrations (46.24%). Due to contact of the boiling water with the plant material, boiling has the highest leaching compared to the other cooking methods and thus leads to a higher reduction of tannins and alkaloids than other cooking methods [17,46]. There were similar reductions in tannin, phytate and alkaloid contents observed in leaves of *Momordica balsamina*, *Amaranthus hybrids*, and *Bidens pilosa* after boiling and microwave treatment [12]. However, Yadav and Sehgal [50] reported that cooking did not change the phytic acid content of leaves. This may be because cooking renders endogenous phytates inactive through heat, and they are broken down at high temperatures [50]. Our results coincide with the findings of Essack et al. [17] on the reduction of alkaloid content in raw *Momordica balsamina* leaves when boiled for 15 min. Many traditional leafy vegetables are bitter because of alkaloids [17]. The tannin content in foods reduces their palatability because they have an astringent flavor; thus, cooking the pumpkin leaves may enhance their taste [48]. From all the household cooking methods tested, stir-frying resulted in the least loss of phytates, tannins and alkaloids compared to raw leaves; in contrast, however, stir-frying pumpkin leaves caused a higher loss of phytates and tannins than steaming.

## 3. Materials and Methods

### 3.1. Chemicals

The analytical standards of chlorogenic acid, lutein, β-carotene, zeaxanthin, catechin, rutin, methanol acetic acid, ethanol, acetone, hexane, methanol, dimethylsulfoxide, butylated hydroxytoluene (BHT), sodium sulphate, Whatman filter paper number 1, isopropyl alcohol, *N*-hexane, potassium ferricyanide, 2,2-diphenyl-2-picrylhydrazyl (DPPH), potassium persulphate, ABTS, potassium dihydrogen orthophosphate, 5 mM p-nitrophenyl-α-d-glucopyranoside, sodium chloride, amylase enzyme, starch, acarbose, Folin–Ciocalteu reagent, sodium carbonate, ammonium hydroxide, acetic acid, 3-(4, 5-dimethylthiazol-2-yl)-2, 5-diphenyltetrazolium bromide, hydrogen peroxide, acetone and hexane, sodium acetate, 2,4,6-Tris(2-pyridyl)-1,3,5-triazine (TPTZ), hydrochloric acid, ferric chloride and Trolox were purchased from Sigma Aldrich, Johannesburg, South Africa.

### 3.2. Plant Material

As described in Mashiane et al. [14], we prepared African pumpkin leaves for cooking in this study. Following 95 days of propagation, disease- and decay-free African pumpkin and pumpkin leaves (20 kg) were harvested twice from the Zithobeni community’s vegetable garden (Bronkhorstspruit, South Africa, 25.8084° S, 28.7081° E) in 2019. The temperature of the region during summer ranges from 25 to 28 °C during daytime, and the night temperature varies between 15 to 17 °C. The mean annual rainfall is 625 mm. The soil type is red apedal sandy loam. After cleaning the leaves in running water, they were transported to the laboratory in cooler boxes at 10 °C within 2 h and stored at 5 °C in the cold room for 24 h prior to processing. Leaf dimensions were approximately 5 × 14.5 × 21 cm. The leaves were chopped into small pieces of about 2.0 cm in diameter and mixed well to ensure homogeneity. Every cooking technique included 30 replicates, and each replicate sample weighed 100 g.

### 3.3. Household Cooking Techniques

The timing for each household cooking technique was set based on interviews conducted with the people from the Zithobeni area and literature-based evidence. The African pumpkin leaves were cooked using the method described by Mashiane et al. [14].

Boiling: To mimic the traditional method of boiling leaves, we heated 100 g of leaves (in 150 mL of water) at 98 °C on a slow flame for 15 min in a covered stainless-steel pot. The leaves were drained once cooked and rapidly cooled on ice.

Steaming: Leaf samples (100 g) were steamed for 15 min in a stainless-steel steamer pot (98 °C) (Concord 30 CM Stainless Steel Steamer Pot, Los Angles, CA, USA), using 250 mL of boiling water and then quickly cooled on ice.

Microwaving: Using a microwave oven (DefyTM–household, model DMO368 MWM 2030M, Beijing, China) set to 2450 MHz 900 W, 100 g of leaves were placed in a glass dish with 12 mL of water for 15 min. The leaves were removed from the microwave, and the surface temperature was recorded. Thereafter, the samples had the remaining water drained and then cooled on ice.

Stir-frying: After adding olive oil (10 mL) (Wilson’s Extra Virgin Olive oil, Capetown, South Africa) to a preheated pan, 100 g of vegetables were stir-fried for 1–2 min. While the vegetables were stir-frying, the oil reached 130 °C, and the surface temperature of the vegetables was 100 °C. Putting the samples on ice cooled them rapidly. 

A food thermometer probe measures the temperature during the different household cooking techniques (Mingle Development Co., Ltd., Shenzhen, China) [14]. A total of 10 replicate samples (weighing 100 g each) of each cooking technique and 10 replicate samples of raw fresh leaves (control) were freeze-dried (VirTis Sp Scientific, Model # 2kBTES-55, Gardiner, NY, USA) and stored at –80 °C in an Elcold freezer 311 (Type Lab 31, Hobro, Denmark, −85 °C freezer) for further biochemical analysis.

### 3.4. Quantification of Phenolic Metabolites

The samples (50 mg) of freeze-dried (VirTis Sp Scientific, Model # 2kBTES-55, Gardiner, NY, USA) African pumpkin leaves were extracted in 1.5 mL of ethanol/water solution (70:30, *v*/*v*), ultrasonically agitated (MRC ultrasonic cleaning bath model (DC-150H, Netanya, Israel) with 150 W of power, 4.5 L capacity, and 43 kHz of ultrasonic frequency) at 25 °C for 30 min, followed by centrifugation (Hermle Z326k, Hermle Labortechnik GmbH, Wehingen, Germany) at 1000× *g* for 20 min at 4 °C. Following the extraction of the phenolic metabolites, the supernatants were pooled and filtered through a polytetrafluorethylene filter before UPLC-QTOF/MS analysis was conducted. Phenolic metabolites were characterised and quantified by an ultra-performance liquid chromatograph (UPLC) with a Waters Acquity photodiode array detector (PDA) coupled to a Synapt G2 quadrupole time-of-flight (QTOF) mass spectrometer (MS) (Waters, Milford, MA, USA), as described by Managa et al. [16] and Mashitoa et al. [21], without any modifications. The tentative identification of phenolic compounds was based on comparison of MS and MS/MS fragmentation patterns of compounds in the extracts with those of known compounds in literature. The reference calibrants (µg mL^−1^) catechin (LOD 1.4, LOQ 4.2), epicatechin (LOD 5.105, LOQ 15.469) and rutin (LOD 3.294, LOQ 9.981) were used to quantify the compounds based on the extracted areas from their mass chromatograms as described previously by Managa et al. [12] and Mashitoa et al. [19]. The concentrations of phenolic compounds were reported as mg kg^−1^ dry weight basis.

Using UPLC-Q-TOF/MS data, a principal component analysis (PCA) analysed the differences between phenolic metabolic profiles of African pumpkin leaves prepared using different household cooking methods. In this study, the use of PCA was to reduce the number of variables in the data matrix to determine the most discriminating method of cooking African pumpkin leaves at home based on the phenolic metabolites of the leaves. Thus, the UPLC-QTOF/MS data were exported to MetaboAnalyst 5.0 for PCA analysis. An orthogonal projection to latent structure discriminant analysis (OPLS-DA) was performed to identify the potential markers (bioactive metabolites) responsible for the differences between the different household cooking methods of African pumpkin leaves.

### 3.5. Determination of Carotenoids

Carotenoids were determined according to the method of Panfili et al. [51], with a few changes. The β-carotene content was extracted from the freeze-dried leaves (2 g) using 5 mL of acetone:hexane (1:1) with 0.1% butylated hydroxytoluene (BHT) solution in tightly closed tubes held in dark condition. The mixture was separated using a centrifuge (Hermle Labortechnik, Germany Type 2326K, Baden-Württemberg, Germany) at 3450× *g* for 15 min at 25 °C. Afterwards, the residue was rinsed with 3 additional 5 mL volumes of the extraction solvent, and centrifugation took place as before. Thereafter, the supernatants were pooled together, dried with anhydrous sodium sulphate, and then filtered with Whatman filter paper (No 1) and evaporated to dryness under a stream of nitrogen. The extract was re-dissolved in 1 mL of isopropyl alcohol (10%) in n-hexane; this was then stored at 5 °C until further analysis. A Shimadzu Prominence-i-LC-2030C 3D AutoSampler (SIL-20A) HPLC system (Shimadzu, Kyoto, Japan), coupled to a diode array detector, was used for the HPLC analysis. Separation occurred on a Shim Pack GIST column (Sydney, Australia) with dimensions 250 mm × 4.6 mm i.d., 5 µm particle size. The identification of the carotenoids was performed by comparison of retention times and absorption spectra of the compounds in the extract with those of pure standards. The reference calibrants (µg mL^−1^) β-carotene (LOQ 6.32, LOD 19.16), lutein (LOQ 11.71, LOD 35.50) and zeaxanthin (LOQ 14.63, LOD 44.33) quantified the compounds based on area. Results were expressed as mg 100 g^−1^ of dried plant material.

### 3.6. Antioxidant Capacity

The antioxidant capacity was determined through methods previously described by Mashitoa et al. [14] and Seke et al. [52], using a 2,2-diphenyl-2-picrylhydrazyl (DPPH) radical scavenging essay. The reaction mixture contained a 250 µL DPPH (90 µM) solution and 28 µL of the ethanol/water (70:30) extract in a 96-well microplate. The reading of absorbance was at 517 nm, with the results expressed as IC_50_ (mg mL^−1^). The production of the ABTS radical cation (ABTS^+^) was determined by the reaction of the ABTS stock solution (7 mM) with 4.9 mM potassium persulphate at the ratio of 1:1 and leaving the mixture to stand in the dark at 25 °C for 12–16 h before use. An aliquot of 40 µL of the sample at different concentrations was pipetted into 200 µL of the ABTS+ solution. The mixture, protected from light, was incubated in a 96-well microplate reader (SpectraMax M5, Molecular Devices, San Jose, CA, USA). at 37 °C for 10 min; the decrease in absorbance at 734 nm was measured and expressed as IC_50_ (mg mL^−1^).

### 3.7. Antidiabetic Activity

#### 3.7.1. α-Glucosidase Inhibitory Activity

The α-glucosidase inhibitory activity of ethanol/water (70:30) extracts of cooked African pumpkin leaves was determined using the method described by Seke et al. [52]. Briefly, an aliquot of 50 µL of sample and 100 µL of 0.1 M phosphate buffer (pH 6.9) containing glucosidase solution (1 U mL^−1^) mixture was incubated in 96-well plates at 25 °C for 10 min. A 50 µL aliquot of 5 mM p-nitrophenyl-α-d-glucopyranoside solution in 0.1 M phosphate buffer (pH 6.9) was added to each well at 1-min intervals. The reaction mixtures were incubated at 25 °C for 5 min, and the absorbance was measured at 405 nm. The inhibiting activity of α-glucosidase was determined in accordance with the method of Seke et al. [52] and expressed in terms of the IC_50_ (mg mL^−1^) value (i.e., there was 50% inhibition of maximal activity observed with a concentration of African pumpkin leaf extract derived from different cooking techniques). 

#### 3.7.2. α-Amylase Inhibitory Activity

An α-amylase inhibition assay was performed using porcine pancreatic α-amylase, as described previously by Moloto et al. [13]. Briefly, a sample (leaf extract 500 μL) and buffer (500 μL, 0.02 M sodium phosphate buffer, pH 6.9) with sodium chloride (0.006 M) containing amylase solution (0.5 mg mL^−1^) were incubated at 25 °C for 10 min in a 96-well microplate. A 500 µL aliquot of 1% starch solution in 0.02 M sodium phosphate buffer (pH 6.9 with 0.006 M sodium chloride) was added then incubated at 25 °C for 10 min. The addition of KAT amylase reagent to 100 µL of reaction solution for 5 mins was to stop the reaction. After diluting, the mixture’s absorbance was measured at 540 nm (multiplate reader, BMG LABTECH, SpectroStar Nano, Ortenberg, Germany). Acarbose and dimethylsulphoxide (100 µL) without the leaf extract served as controls. Determination of α-amylase inhibition was done by calculating the IC_50_ (mg mL^−1^) value.

### 3.8. Antinutritive Compounds

The tannin content of raw and cooked African pumpkin leaves was determined by using modified vanillin-HCl in methanol according to the method described by Price et al. [53]. Leaf samples (0.2 g each) were mixed with 1% HCl (10 mL) and vortexed vigorously for 20 min. The mixture was centrifuged at 2500× *g* in an M2 rotor model HermLe Z326k (HermLe Labortechnik GmbH, Wehingen, Germany) for 5 min at 15° C. The supernatant was pipetted and mixed with 50 µL of vanillin-HCl in methanol (5 mL of 8% HCl in methanol and 1% vanillin in methanol (5 mL)) in a microplate and incubated at 30 °C for 20 min. A standard curve was constructed using a series of tannic acids at different concentrations and thereafter by measuring the absorbance at 500 nm.

The phytate content was determined using Wade’s reagent [16]. A 2 mL aliquot of 2.4% HCl was added to 100 mg of sample in an Eppendorf tube, sonicated (MRC ultrasonic cleaning bath model (DC-150H, Netanya, Israel) with 150 W of power, 4.5 L capacity, and 43 kHz of ultrasonic frequency) at 25 °C for 20 min then centrifuged (Hermle Z326k, Hermle Labortechnik, Baden-Württemberg, Germany) at 2000× *g* for 15 min at 4 °C. Wade’s reagent (133 µL) was added to the supernatant (200 µL) and the absorbance was measured at 500 nm using a microplate reader (Spectrostar Nano BMG LABTECH, SpectroStar Nano, Ortenberg, Germany). The phytate content was expressed as mg 100 g^−1^ using phytic acid sodium salt as a standard. The alkaloid content was determined by NH_4_OH precipitation [16]. The vegetable sample (0.1 g) was mixed with a 2 mL aliquot of 10% acetic acid in ethanol (*v*/*v*) and sonicated for 1 h at 25 °C, and the mixture was centrifuged at 2000× *g* for 15 min at 4 °C (Hermle Z326k, Hermle Labortechnik GmbH, Baden-Württemberg, Germany). The collected supernatant had concentrated ammonium hydroxide added to it dropwise until the precipitation stopped; the solution was left to stand for the precipitation to settle. After collecting the precipitate, it was washed with distilled water and ammonium hydroxide (*v*/*v*). The remaining residue was left to dry at room temperature and weighed, with the results expressed as mg per 100 g of dried material.

### 3.9. Statistical Analysis

This study employed a completely randomised design with five replicates per cooking technique for antioxidant capacity, antidiabetic enzymes and antinutrient compounds, and the experiment was repeated twice. For the UPLC analysis, three replicates per treatment were used, and the data were imported for performing partial least squares discriminant analysis (PLS-DA), variable importance in projection (VIP) scores, and heat maps.

The Genstat (VSN International, Hemel Hempstead, UK) for Windows 13th Edition (2010 version) analysed the differences between household cooking techniques using a one-way ANOVA. To compare the means of the cooking treatments, the least significant difference test (LSD) was used, with *p* < 0.05. Results were imported into MetaAnalyst 5.0, for performing partial least squares discriminant analysis (PLS-DA), variables importance in projection (VIP) scores, and heat maps.

## 4. Conclusions

The study demonstrated that household cooking techniques considerably influence the bioactive metabolites, carotenoids, antioxidant activity, antidiabetic activity and antinutrient intake of African pumpkin leaves. Out of all evaluated household cooking methods stir-frying improved the availability of phenolic compounds and retained the carotenoid components. The correlation analysis identified the biological activities in stir-fried leaves that are responsible for these functional properties. However, boiling greatly reduced the antinutrient components from the leaves. Ultimately, stir-frying can be recommended as a suitable household cooking method for African pumpkin leaves based on the results of this study.

## Figures and Tables

**Table 1:** Impact of different household cooking techniques on the bioactive metabolites of African pumpkin leaves (*Momordica balsamina* L.).

Metabolites (mg kg^−1^)	Raw Leaves	Steaming	Microwaving	Boiling	Stir-Frying
Methylquinic acid	3.96 ± 0.71 *^a^	1.44 ± 0.26 ^c^	2.53 ± 3.29 ^b^	2.08 ± 0.99 ^b^	0.33 ± 0.06 ^d^
4-caffeoylquinicacid (cryptochlorogenic acid)	6.61 ± 1.18 ^d^	76.01 ± 13.57 ^b^	3.34 ± 2.13 ^e^	8.35 ± 1.36 ^c^	507.02 ± 90.54 ^a^
*cis*-4-coumaroylquinic acid	33.04 ± 1.90 ^b^	12.17 ± 2.17 ^d^	1.47 ± 0.44 ^e^	20.82 ± 5.81 ^c^	99.83 ± 17.83 ^a^
*trans*-4-coumaroylquinic acid	17.50 ± 3.12 ^b^	13.61 ± 2.43 ^c^	1.57 ± 0.33 ^e^	10.34 ± 5.60 ^d^	116.57 ± 20.82 ^a^
*cis*-4-feruloylquinic acid	1505.20 ± 2.7 ^a^	261.19 ± 46.6 ^d^	21.87 ± 2.98 ^e^	299.05 ± 74.2 ^c^	983.1 ± 175.56 ^b^
Trans-4-feruloylquinic acid	244.86 ± 4.72 ^a^	19.54 ± 3.49 ^b^	1.38 ± 0.71 ^d^	6.06 ± 4.31 ^c^	259.99 ± 46.43 ^a^
Quercetin-3-galactoside	22.26 ± 3.97 ^b^	15.59 ± 2.78 ^c^	0.62 ± 0.32 ^e^	1.33 ± 0.92 ^d^	206.06 ± 36.80 ^a^
Phenethyl rutinoside	95.73 ± 17.10 ^a^	10.04 ± 1.79 ^c^	1.13 ± 0.28 ^d^	11.96 ± 1.52 ^c^	60.90 ± 10.87 ^b^
Rhamnetin-3-*O*-glucoside	5.76 ± 1.03 ^b^	2.56 ± 0.46 ^c^	0.17 ± 0.18 ^d^	0.27 ± 0.10 ^d^	55.43 ± 9.90 ^a^
Quercetin-3-rutinoside (rutin)	175.24 ± 3.29 ^c^	209.68 ± 37.4 ^b^	7.94 ± 2.41 ^d^	8.15 ± 1.49 ^d^	405.15 ± 72.35 ^a^
Pseudolaroside A	246.97 ± 1.10 ^b^	155.12 ± 27.7 ^c^	5.45 ± 0.62 ^e^	81.31 ± 24.09 ^d^	678.89 ± 121.2 ^a^
β-d-glucosyl-2-coumarate (melilotoside)	15.12 ± 1.70 ^c^	31.71 ± 5.66 ^a^	1.34 ± 0.54 ^e^	17.37 ± 1.60 ^b^	6.10 ± 1.09 ^d^
Kaempferol-*O*-rutinoside (nicotiflorin)	231.27 ± 1.30 ^b^	50.76 ± 9.07 ^c^	0.94 ± 0.69 ^e^	1.80 ± 0.40 ^d^	425.81 ± 76.04 ^a^
Isorhamnetin-3-*O*-robinoside (keioside)	41.76 ± 1.46 ^b^	12.49 ± 2.23 ^c^	0.52 ± 0.42 ^d^	0.61 ± 0.33 ^d^	249.26 ± 44.51 ^a^

* Standard deviation, the Fisher’s least significant difference (LSD) test shows that means in the same row with different superscript alphabetic letter letters a–e is significantly different (*p* ≤ 0.05).

**Table 2:** The effects of different household cooking techniques on carotenoid components of African pumpkin leaves (*Momordica balsamina* L.).

Household Cooking Methods	Lutein mg 100 g^−1^	% Loss or Gain	Zeaxanthin mg 100 g^−1^	% Loss or Gain	β-Carotene mg 100 g^−1^	% Loss or Gain	Total Carotenoids
Raw	34.13 ± 0.45 *^c^		1.30 ± 0.20 ^bc^		9.05 ± 0.01 ^d^		44.48 ^d^
Stir-frying	54.69 ± 1.0 ^a^	60.24 ± 0.53 ^a^	3.20 ± 0.29 ^a^	146.15 ± 0.62 ^a^	20.68 ± 0.10 ^a^	123.51 ± 0.70 ^a^	78.57 ^a^
Boiling	43.08 ± 1.8 ^b^	26.22 ± 0.20 ^c^	2.25 ± 0.25 ^ab^	73.08 ± 0.75 ^b^	12.41 ± 0.08^c^	37.13±0.52 ^c^	57.74 ^c^
Steaming	44.51 ± 1.1 ^b^	30.41 ± 0.60 ^b^	1.85 ± 1.24 ^b^	42.31 ± 0.64 ^d^	17.33 ± 0.03 ^b^	91.49 ± 0.43 ^b^	63.69 ^bc^
Microwave	41.71 ± 1.2 ^b^	22.21 ± 0.33 ^c^	0.64 ± 0.20 ^c^	50.77 ± 0.54 ^c^	7.97 ± 0.03 ^e^	11.93 ± 0.54 ^d^	50.32 ^cd^

* Standard deviation, the Fisher’s least significant difference (LSD) test shows that means in the same row with different superscript alphabetic letter letters a–e is significantly different (*p* ≤ 0.05), different alphabet letters in the same column for African pumpkin leaves indicate significant differences at *p* < 0.05.

**Table 3:** Effects of household cooking techniques on antioxidant and antidiabetic activities of African pumpkin leaves (*Momordica balsamina* L.).

Household Cooking Techniques	DPPH IC_50_ mg mL^−1^	ABTS IC_50_ mg mL^−1^	α-Glucosidase IC_50_ mg mL^−1^	α-Amylase IC_50_ mg mL^−1^
Raw	1.78 ± 0.10 *^d^	0.78 ± 0.02 ^d^	2.23 ± 0.20 ^d^	1.31 ± 0.21 ^d^
Stir-frying	0.71 ± 0.20 ^e^	0.61 ± 0.04 ^e^	1.35 ± 0.09 ^e^	0.80 ± 0.16 ^e^
Microwave	4.16 ± 0.08 ^a^	3.52 ± 0.25 ^a^	5.97 ± 0.79 ^a^	4.64 ± 0.20 ^a^
Steaming	2.62 ± 0.11 ^c^	1.21 ± 0.03 ^c^	3.28 ± 0.28 ^c^	2.58 ± 0.25 ^c^
Boiling	3.82 ± 0.25 ^b^	2.62 ± 0.11 ^b^	4.14 ± 0.00 ^b^	3.81 ± 0.11 ^b^
Acarbose			6.87 ± 0.22	3.14 ± 0.13

* Standard deviation. the Fisher’s least significant difference (LSD) test shows that means in the same row with different superscript alphabetic letter letters a–e is significantly different (*p* ≤ 0.05).

**Table 4:** Effects of household cooking techniques on antinutritive compounds present in African pumpkin leaves (*Momordica balsamina* L.).

Household Cooking Techniques	Tannins mg 100 g^−1^	% Loss	Phytates mg 100 g^−1^	% Loss	Alkaloids mg 100 g^−1^	% Loss
Raw	165.20±1.68 *^a^		41.37 ± 0.36 ^a^		35.00 ± 0.05 ^a^	
Stir fry	157.31 ± 0.45 ^b^	5.88 ±0.52 ^c^	33.00 ± 1.40 ^b^	20.23 ± 0.18 ^c^	23.60 ± 0.29 ^b^	32.57 ± 0.53 ^d^
Boiling	56.43 ± 1.26 ^e^	66.25 ± 0.17 ^a^	18.41 ± 0.53 ^d^	55.49 ± 0.28 ^a^	4.04 ± 0.08 ^e^	88.46 ± 0.26 ^a^
Steaming	131.85 ± 0.99 ^c^	21.14 ± 0.17 ^b^	28.75 ± 1.03 ^c^	30.51 ± 0.39 ^b^	17.73 ± 0.28 ^c^	49.34 ± 0.47 ^c^
Microwave	63.48 ± 1.18 ^d^	62.03 ± 0.08 ^a^	20.15 ± 0.38 ^d^	51.29 ± 0.44 ^a^	14.88 ± 0.29 ^d^	57.49 ± 0.45 ^b^

* Standard deviation, the Fisher’s least significant difference (LSD) test shows that means in the same row with different superscript alphabetic letter letters a–e is significantly different (*p* ≤ 0.05).

## Data Availability

All data obtained during this study are presented in the manuscript and also in Supplementary Figures S1–S14.

## Supplementary Materials

The following supporting information can be downloaded at: https://www.mdpi.com/article/10.3390/molecules27061901/s1, Supplementary Figures S1–S14 provides the UV spectrum, MS and MS/MS fragmentation pattern of the identified phenolic molecules.
