# Supplementary material for: A Comparison of Bioactive Metabolites, Antinutrients, and Bioactivities of African Pumpkin Leaves (Momordica balsamina L.) Cooked by Different Culinary Techniques
Source: Molecules. 2022 Mar 15;27(6):1901. doi: 10.3390/molecules27061901 (PMC8951283; doi:10.3390/molecules27061901)
Supplement: Supplementary file 1 [file molecules-27-01901-s001.zip › molecules-1606308-supplementary.pdf]

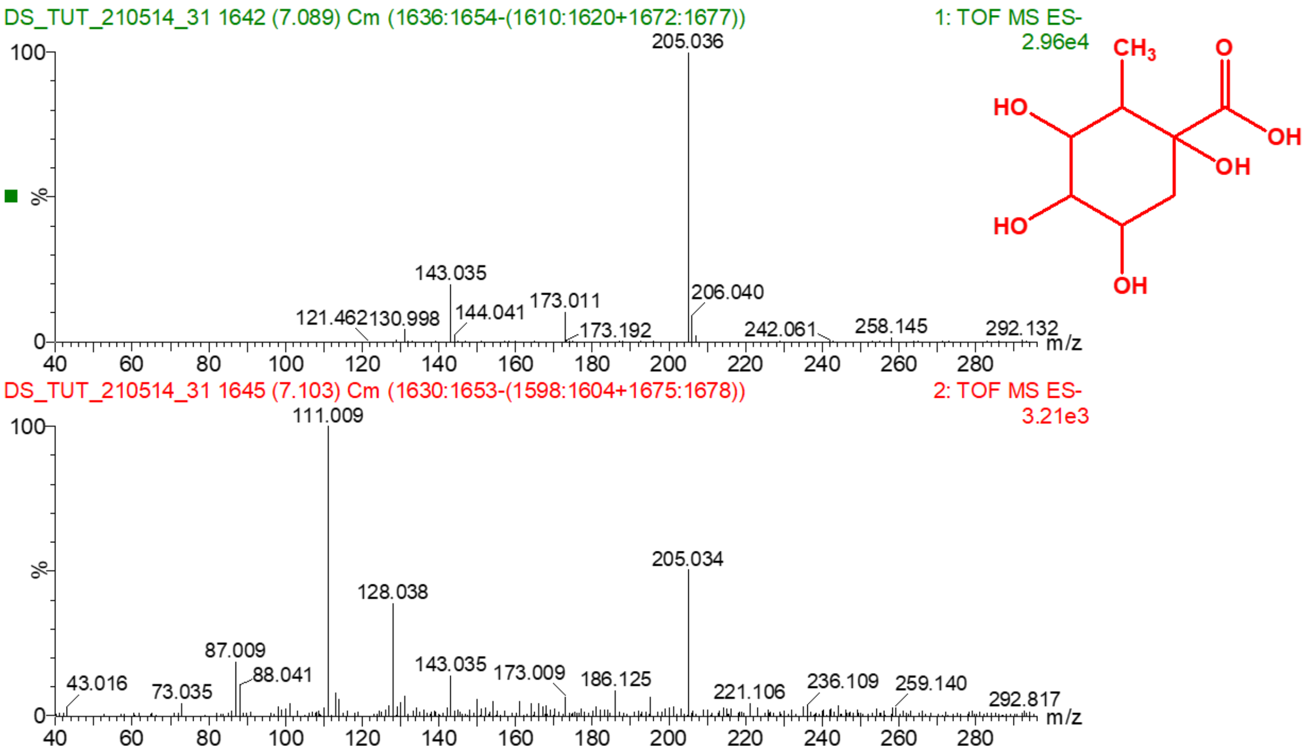

Figure S1. Showing the chemical structure of methyl quinic acid superimposed on an overlay of its MS and MS/MS fragmentation pattern.

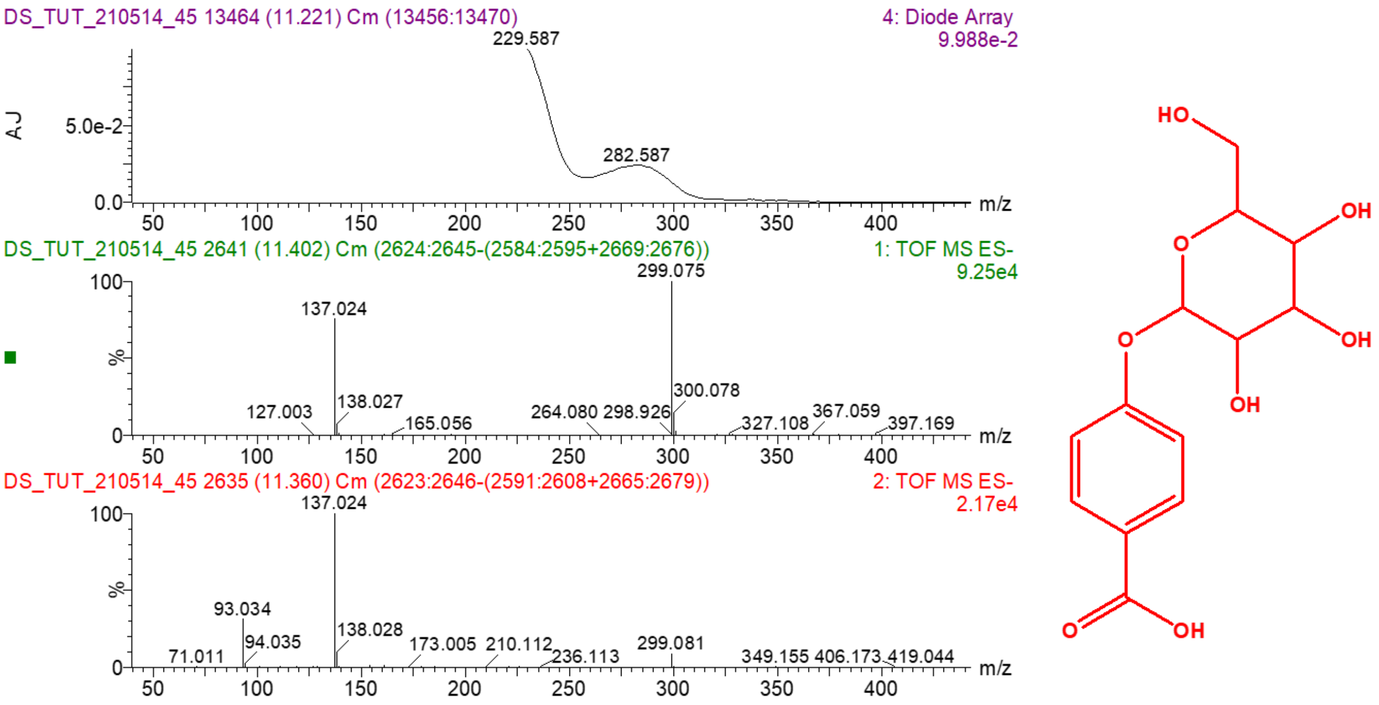

Figure S2. Showing an overlay of the UV spectrum, MS and MS/MS fragmentation pattern of pseudolaroside A next to its chemical structure.

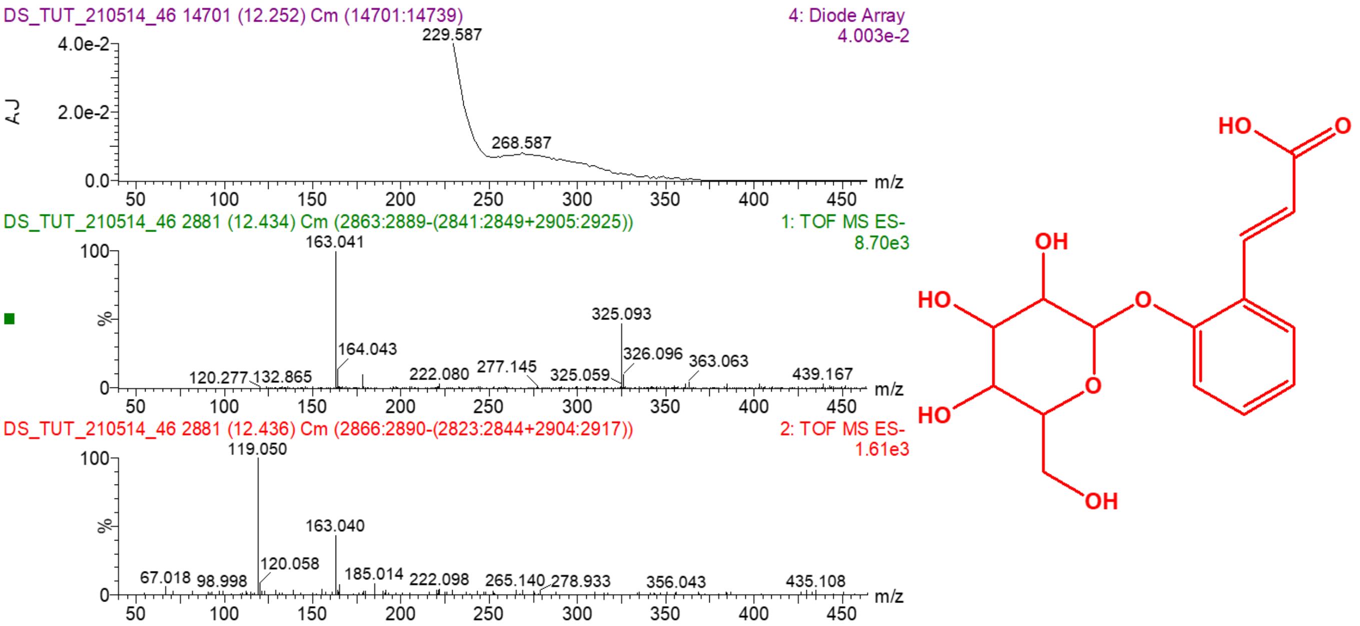

Figure S3. Showing an overlay of the UV spectrum, MS and MS/MS fragmentation pattern of melilotoside next to its chemical structure.

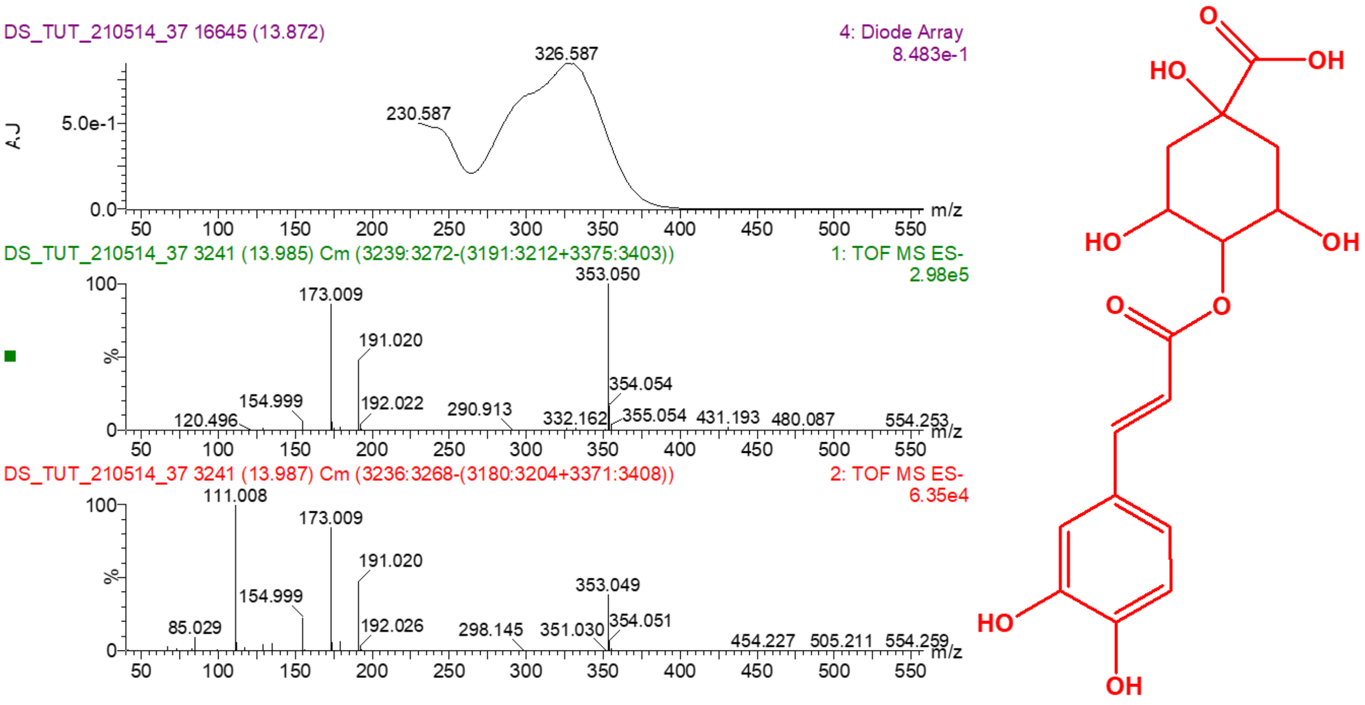

Figure S4. Showing an overlay of the UV spectrum, MS and MS/MS fragmentation pattern of cryptochlorogenic acid next to its chemical structure.

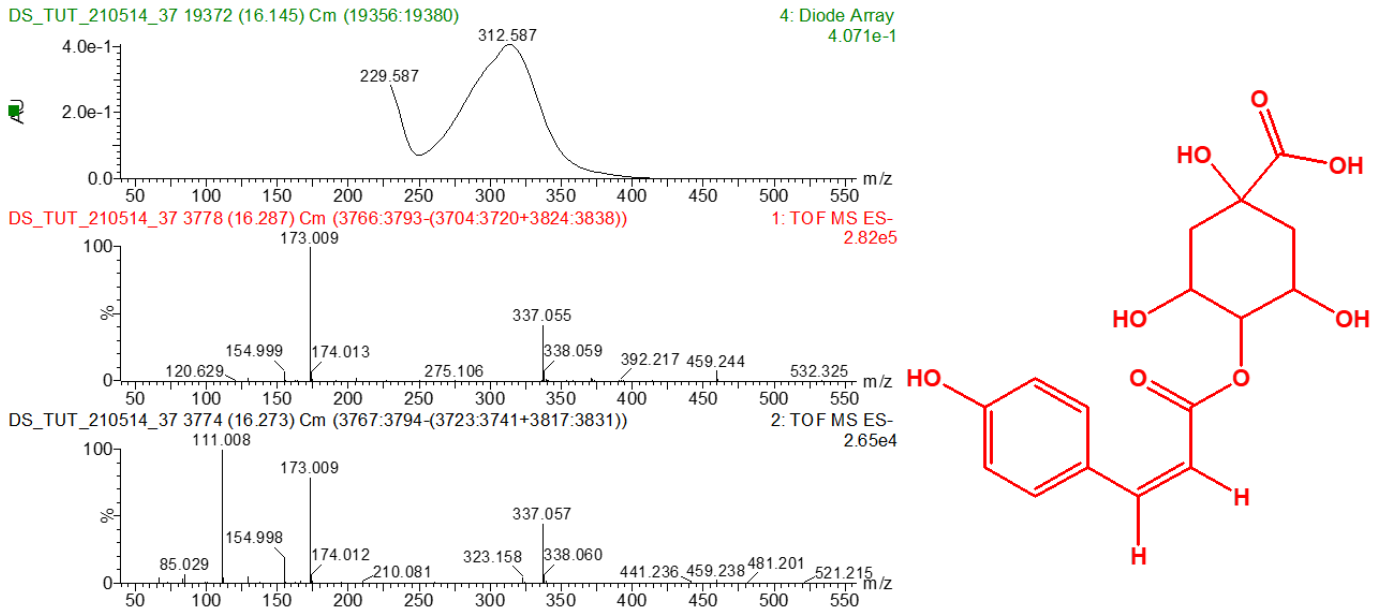

Figure S5. Showing an overlay of the UV spectrum, MS and MS/MS fragmentation pattern for cis-4-coumaroylquinic acid next to its chemical structure.

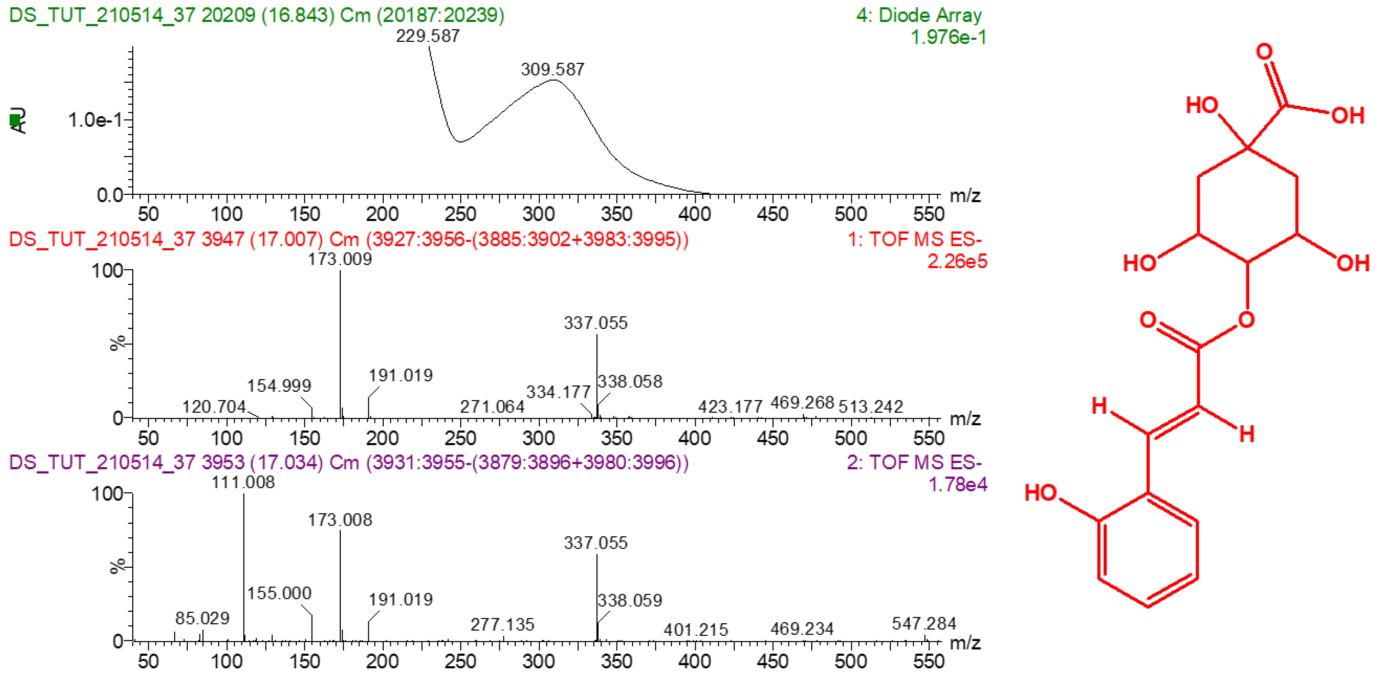

Figure S6. Showing an overlay of the UV spectrum, MS and MS/MS fragmentation pattern for trans-4-coumaroylquinic acid next to its chemical structure.

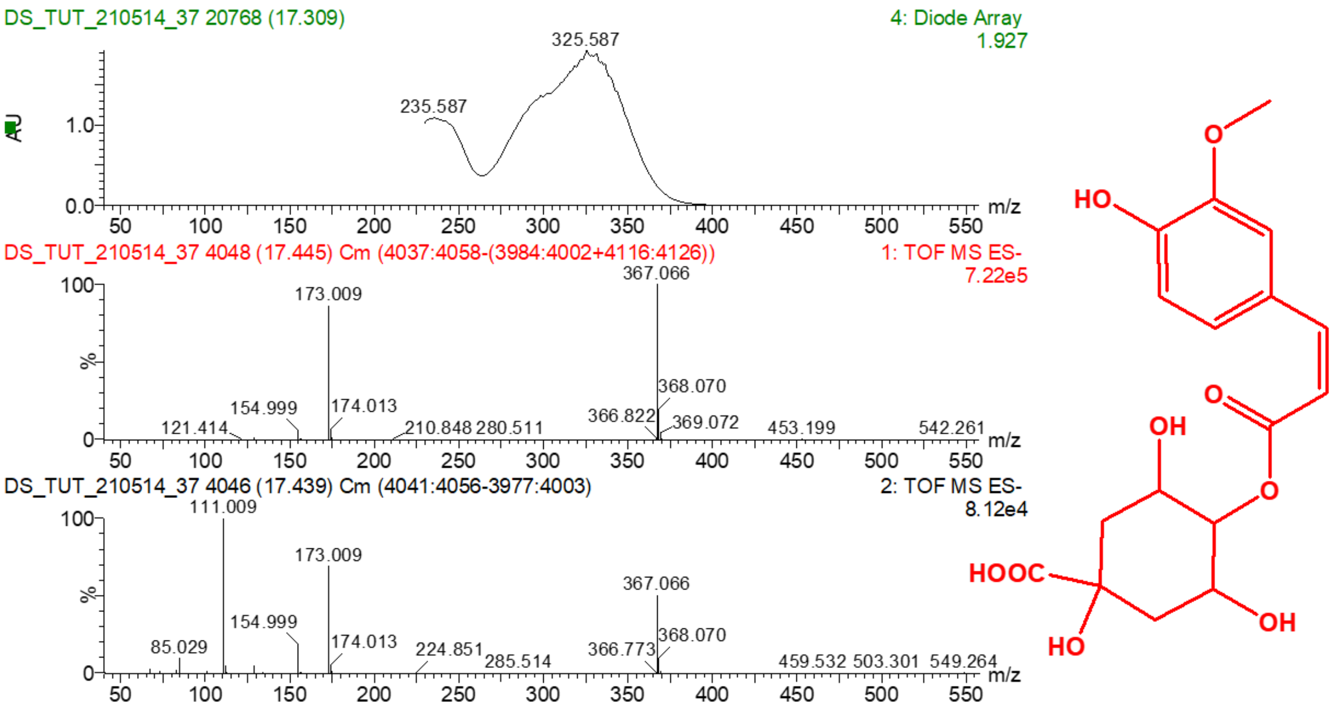

Figure S7. Showing an overlay of the UV spectrum, MS and MS/MS fragmentation pattern for cis-4-feruloylquinic acid next to its chemical structure.

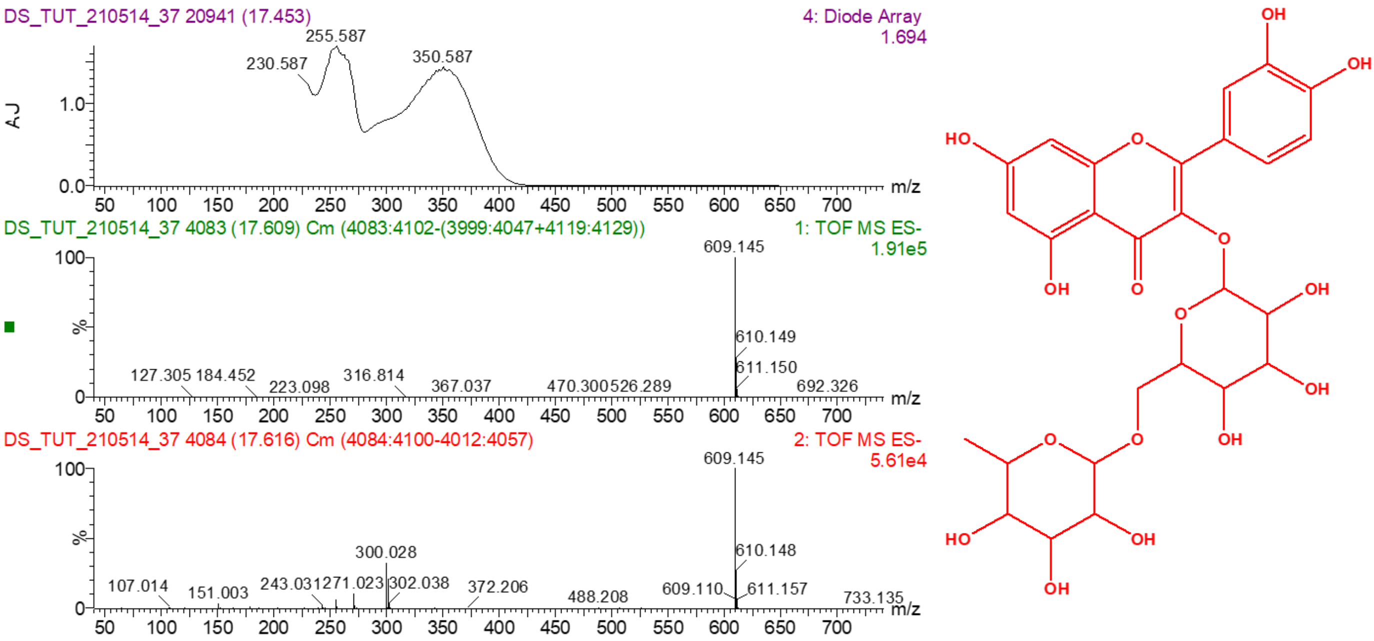

Figure S8. Showing an overlay of the UV spectrum, MS and MS/MS fragmentation pattern of rutin next to its chemical structure.

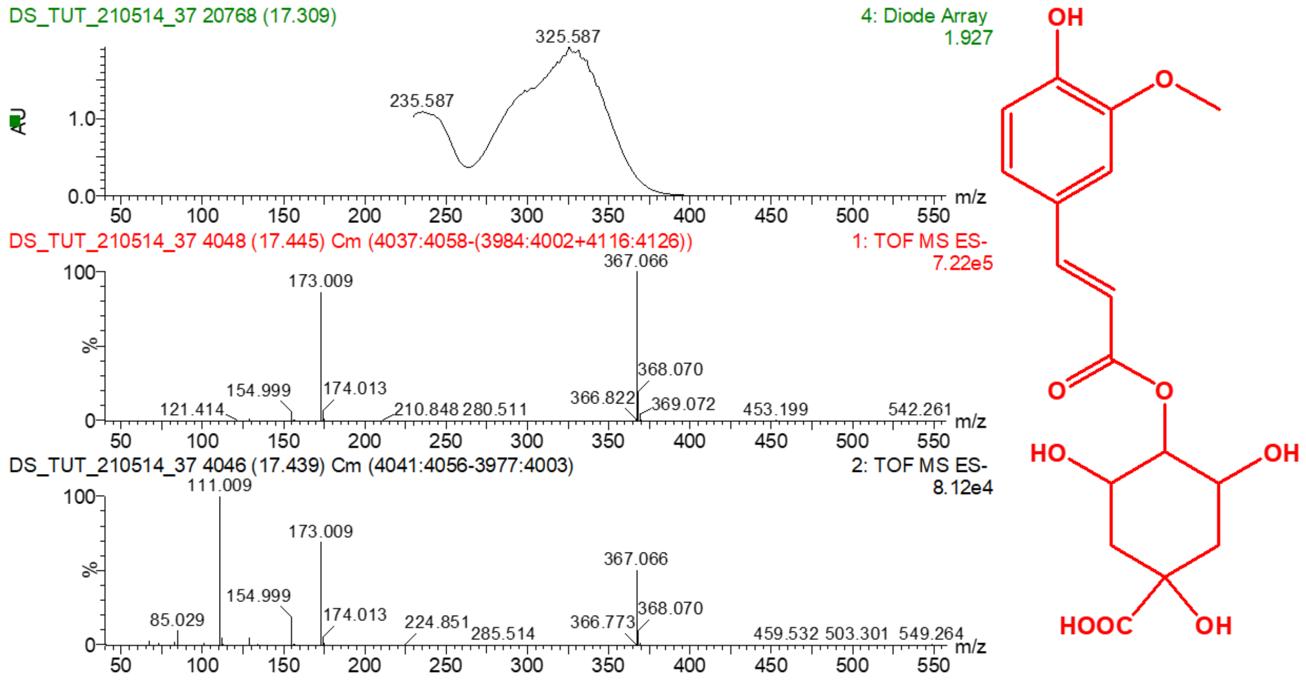

Figure S9. Showing an overlay of the UV spectrum, MS and MS/MS fragmentation pattern of trans 4 feruloylquinic acid next to its chemical structure.

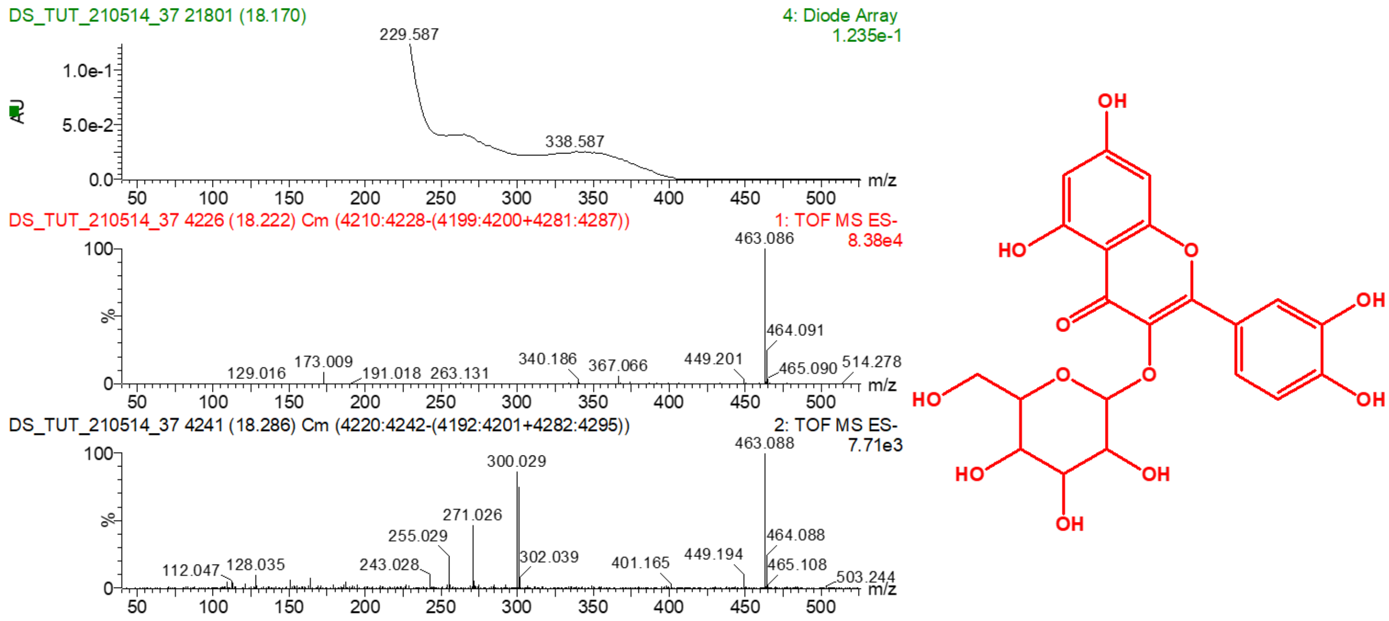

Figure S10. Showing an overlay of the UV spectrum, MS and MS/MS fragmentation pattern of quercetin 3 galactoside next to its chemical structure.

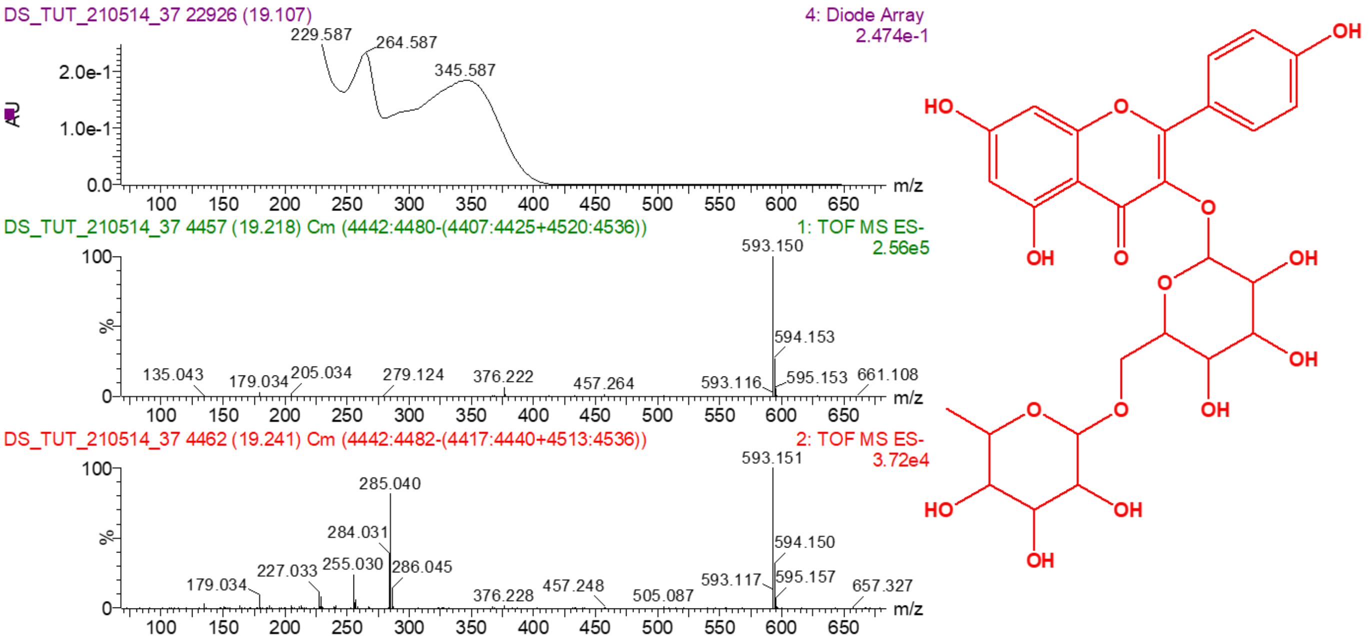

Figure S11. Showing an overlay of the UV spectrum, MS and MS/MS fragmentation pattern of nicotiflorin next to its chemical structure.

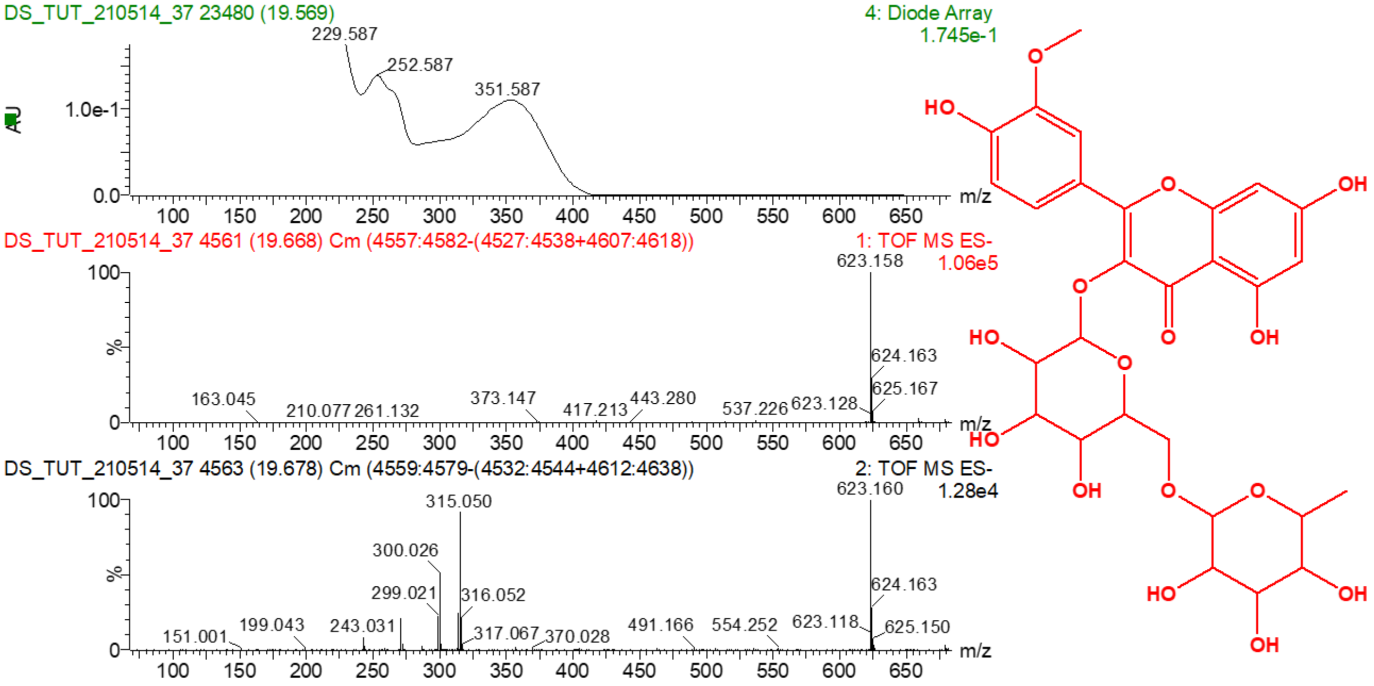

Figure S12. Showing an overlay of the UV spectrum, MS and MS/MS fragmentation pattern of nicotiflorin next to its chemical structure.

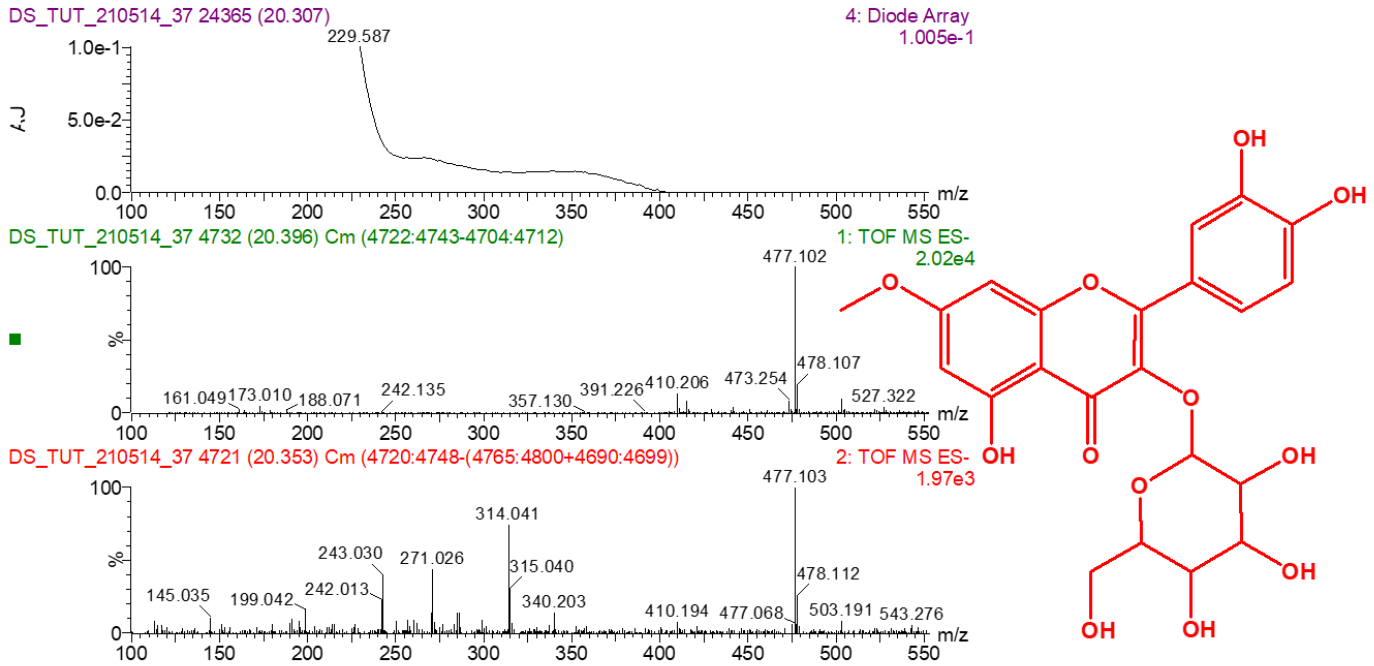

Figure S13. Showing an overlay of the UV spectrum, MS and MS/MS fragmentation pattern of rhamnetin-3-O-glucoside next to its chemical structure.

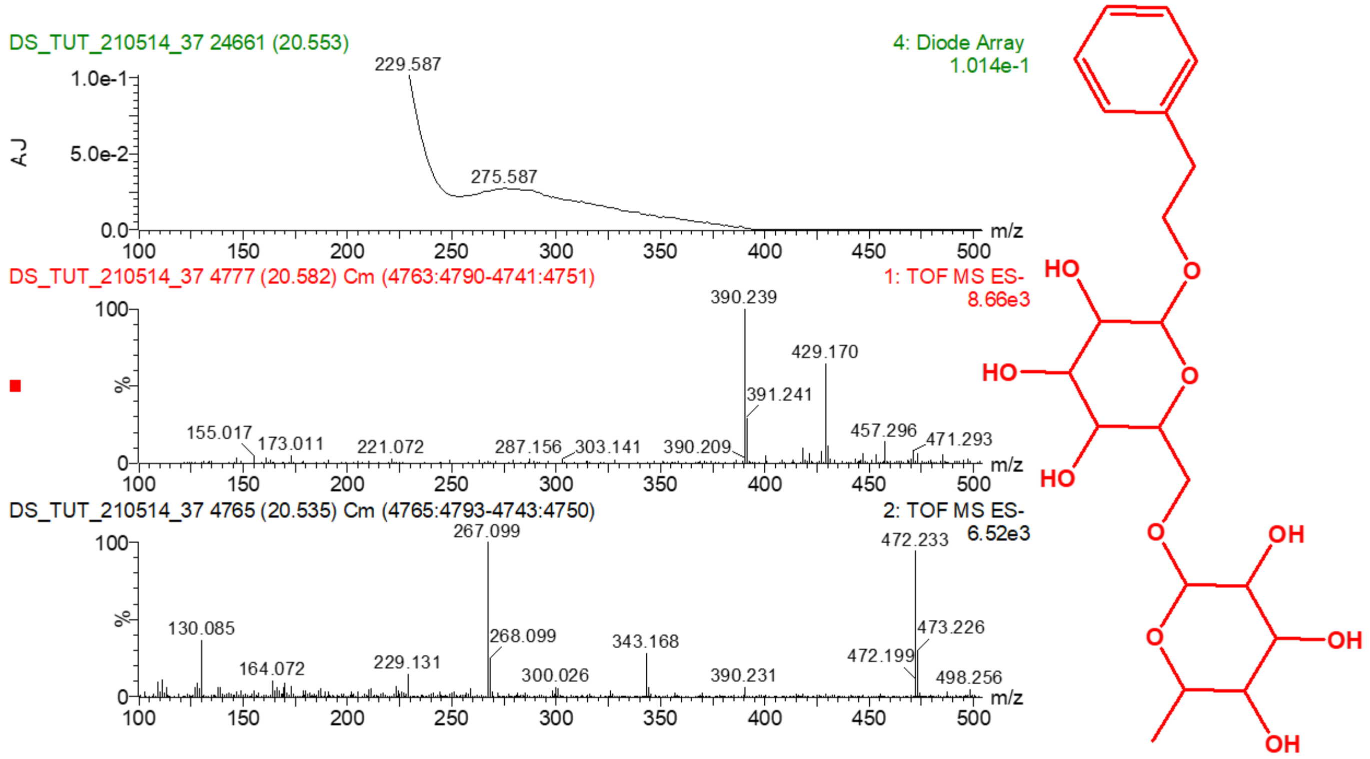

Figure S14. Showing an overlay of the UV spectrum, MS and MS/MS fragmentation pattern of phenethyl rutinoside next to its chemical structure.
